# The highly GABARAP specific rat monoclonal antibody 8H5 visualizes GABARAP in immunofluorescence imaging at endogenous levels

**DOI:** 10.1038/s41598-018-36717-1

**Published:** 2019-01-24

**Authors:** Indra M. Simons, Jeannine Mohrlüder, Regina Feederle, Elisabeth Kremmer, Thomas Zobel, Jochen Dobner, Nicole Bleffert, Silke Hoffmann, Dieter Willbold

**Affiliations:** 1 Institut für Physikalische Biologie, Heinrich-Heine-Universität, 40225 Düsseldorf, Germany; 2 Institute of Complex Systems, Structural Biochemistry (ICS-6), Forschungszentrum Jülich, 52425 Jülich, Germany; 3 Institute for Diabetes and Obesity, Monoclonal Antibody Core Facility, 85764 Neuherberg, Germany; 4 Institute for Molecular Immunology, Helmholtz Center Munich, German Research Center for Environmental Health, 85764 Neuherberg, Germany; 5 Department of Biology, Center for Advanced Imaging (CAi), Heinrich-Heine-Universität, 40225 Düsseldorf, Germany

**Keywords:** Fluorescence imaging, Autophagy

## Abstract

The determination of unique functions of GABARAP (gamma-aminobutyric acid type A receptor-associated protein), a member of the highly conserved protein family of mammalian autophagy-related 8 protein (mATG8), within diverse cellular processes remains challenging. Because available anti-GABARAP antibodies perform inadequate, especially within various microscopy-based applications, we aimed to develop an antibody that targets GABARAP but not its close orthologs. Following the latest recommendations for antibody validation including fluorescence protein tagging, genetic and orthogonal strategies, we characterized the resulting anti-GABARAP (8H5) antibody during confocal immunofluorescence imaging in-depth. We compared the antibody staining pattern with that obtained for fluorescence protein tagged GABARAP, GABARAPL1 or GABARAPL2 each ectopically expressed in GABARAP knockout cells. Furthermore, we imaged cells expressing all mATG8 family members at endogenous levels and checked GABARAP knockout cells for unspecific staining under fed or macroautophagy-inducing conditions. Finally, we simultaneously stained cells for endogenous GABARAP and the common autophagosomal marker LC3B. Summarized, the presented antibody shows high specificity for GABARAP without cross-reactivity to other mATG8 family members in immunofluorescence imaging making it a valuable tool for the identification of unique GABARAP functions.

## Introduction

Autophagy-related 8 (ATG8) proteins form a highly conserved eukaryotic protein family, which generate small-sized products of approximately 15 kDa with high overall structural similarities^1^. Contrary to the situation in yeast which has one single *Atg8* gene, mammalian genomes code for several ATG8 paralogs. The respective proteins are divided in two subfamilies: the microtubule-associated protein 1 light chain 3 (MAP1LC3) subfamily (referred to as LC3s) including LC3A, LC3B, LC3B2, LC3C, and the GABARAP (γ-amino-butyric acid receptor-associated protein) subfamily (referred to as GABARAPs) with GABARAP, GABARAPL1/GEC1 (GABARAP-like 1/Glandular epithelial cell protein) and GABARAPL2/GATE-16 (GABARAP-like 2/Golgi-associated ATPase enhancer of 16 kDa) in humans. Mammalian (m)ATG8s show ubiquitous expression patterns, although for some family members increased expression levels are documented in certain tissues and their expression underlies regulation through various mechanisms^2^.

The broad action spectrum of these adaptor-like proteins with partially overlapping features is far from being completely understood^3^. First described to participate in the trafficking of type-A receptors for the neurotransmitter gamma-aminobutyric acid (GABA) in neurons^4^, GABARAP, the prototype of the GABARAP subfamily, is implicated in a variety of intracellular transport processes including the shipping and correct organization of further receptors^5–7^ as well as in autophagy, an evolutionarily highly conserved process essential for cellular homeostasis^8^. Like all ATG8s, GABARAP can undergo lipidation by an ubiquitin-like conjugation system^9,10^ promoting its association to autophagosomal membranes^11,12^ but likely also to (tubule)vesicular structures in GABARAP-mediated protein trafficking^13^. Meanwhile, functional divergences between the divers ATG8s became obvious, like their action at different stages of autophagosome biogenesis as well as during autophagosomal cargo recruitment, where specific interactions with divers scaffolding proteins or autophagic receptors are formed in selective autophagy^3,14^. Within the mATG8s, LC3B is considered as an established and widely accepted marker for autophagosomes^15^. In this context, recent studies point towards a crucial role of the GABARAP subfamily members especially in autophagosome and lysosome fusion^16^. Under nutrient rich conditions, GABARAP was shown to accumulate in the pericentriolar material from which it can be translocated to forming autophagosomes during starvation^17,18^.

Despite this apparent progress, elucidation of unique roles for individual ATG8 members within a specific process remains to be a challenging task. Although fluorescent protein (FP)-tagged ATG8 reporters, which are widely used to study autophagy as well as ATG8s’ functions, delivered multiple new insights, their application might be accompanied by artifacts resulting from overexpression or steric hindrance due to the bulky FP-tag. In parallel, available antibodies against diverse ATG8 family members often show cross-reactivity, and are frequently not sufficiently validated in a transparent manner for the user, especially regarding their performance within diverse applications. As long as such information is lacking, every ATG8-targeted antibody result has to be interpreted with caution unless specificity of the antibody in use has clearly been demonstrated for the application and the ATG8 member chosen^15^.

In this study, we performed an in-depth characterization of an in-house generated rat monoclonal antibody (mAb) against human (h)GABARAP (anti-GABARAP (8H5) antibody) by taking the latest recommendations for antibody validation into account^19^. Thereby, we focused on immunofluorescence (IF) staining, and included genetic (knockout (KO) cell-based), orthogonal (autophagosome counting under growth factor depleted conditions), tagged-target protein expression and independent antibody-based (commercial anti-GABARAP and anti-LC3B) strategies as validation pillars. Our analysis revealed that the anti-GABARAP (8H5) antibody shows high specificity for GABARAP without cross-reactivity for GABARAPL1, -L2 or LC3 subfamily members in our set-up. With the help of this antibody we investigated the colocalization of GABARAP and LC3B under endogenous conditions. To our knowledge, anti-GABARAP (8H5) antibody outperforms so far available antibody resources during localization studies in fixed cells, and thus reasonably enlarges our current tool box that is needed to identify unique GABARAP activities with high quality and consistency in an unambiguous manner.

## Results

### Anti-GABARAP (8H5) antibody discriminates between purified recombinant GABARAP, -L1, and -L2

**Figure 1:**
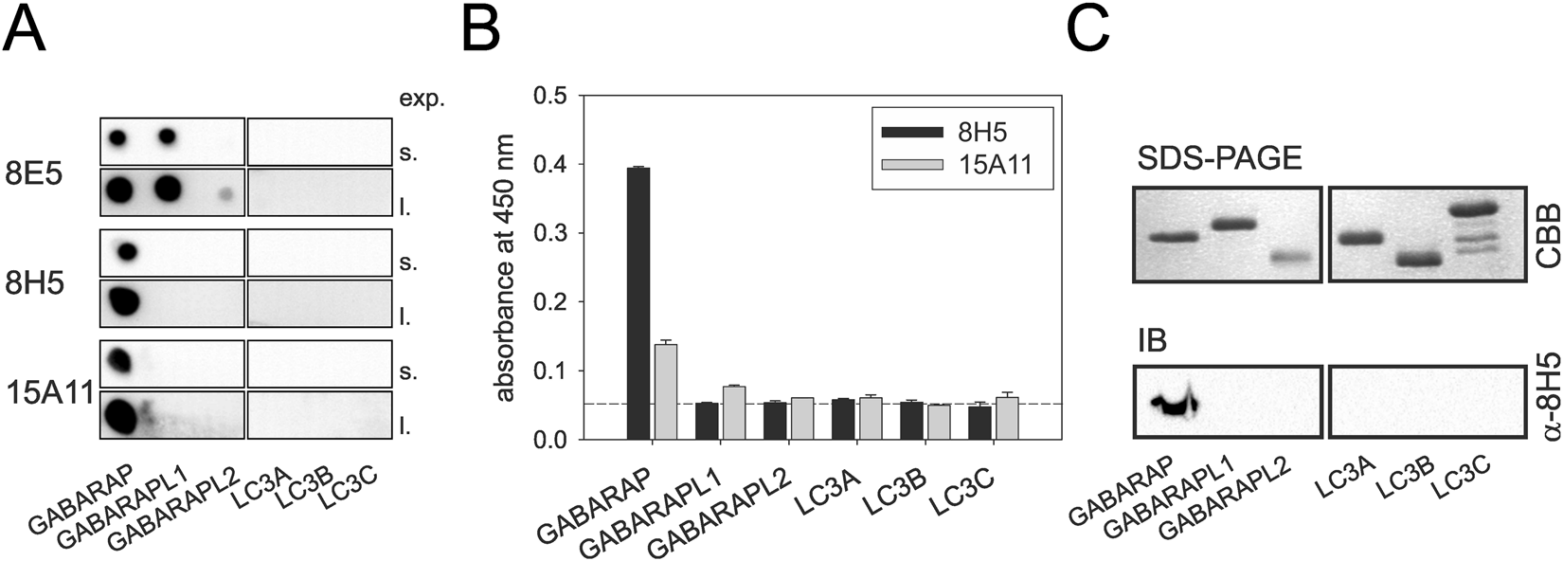
Anti-GABARAP (8H5) antibody does not react with LC3s and discriminates between GABARAP, GABARAPL1 and GABARAPL2. (**A**) Dot blots analysis using three different hybridoma supernatants to detect GABARAP binding and putative cross-reactivity to the other ATG8 family members. Purified recombinant proteins (10 µM each) were transferred to a cellulose nitrate membrane. Short (s.) and long (l.) exposure times of one representative blot each (n = 2) are given. (**B**) ELISA using anti-GABARAP (8H5) and anti-GABARAP (15A11) antibodies to confirm binding specificity and to assess the signal-to-noise ratio as a measure for mAb binding strength. Purified recombinant proteins (700 ng each) were coated on a 96-well plate and were incubated for 1 h at room temperature (RT) before antibody detection. Primary antibody containing hybridoma supernatants anti-GABARAP (8E5) and anti-GABARAP (15A11) and secondary goat anti-rat IgG-HRP antibody were incubated for 1 h at RT, respectively (n = 2). (**C**) Purified recombinant proteins (1 µg each) were subjected to SDS-PAGE in duplicate and were stained with Coomassie brilliant blue (CBB) to visualize the input or were used for immunoblotting with anti-GABARAP (8H5) mAb followed by HRP-coupled mouse anti-rat IgG2a antibody detection. Uncropped versions of (**A**) and (**C**) are given in Supplementary Fig. S3A,B.

In order to generate a monoclonal GABARAP antibody that is able to discriminate GABARAP from its various related ATG8 family members, rats were immunized with full-length hGABARAP fused to GST (glutathione S-transferase) (GST-GABARAP). After fusion of their immune spleen cells with the myeloma cell line P3X63-Ag8.653, the resulting hybridoma supernatants were collected and confirmed to react with immobilized GST-GABARAP by ELISA (enzyme-linked immunosorbent assay) (data not shown). Next, selectivity of in total 38 reacting supernatants was assessed using the dot blot technique. To this end, recombinant purified GABARAP, -L1, -L2 as well as the LC3s A, B, and C were spotted as target. Approximately 80% of the tested GABARAP-reactive supernatants failed this quality check, because, besides GABARAP, they also bound to its closest relative, GABARAPL1, as exemplarily shown for the supernatant corresponding to clonal line 8E5 (Fig. 1A, top panel). A few, among them anti-GABARAP (8H5) and anti-GABARAP (15A11) mAb containing supernatants, showed selective binding to GABARAP demonstrating their high specificity in this application (Fig. 1A, mid and bottom panels). Finally, binding specificity of anti-GABARAP (8H5) and (15A11) antibodies was confirmed by ELISA (Fig. 1B) and western blot analysis (Fig. 1C), respectively. Again, immunoreactivity could exclusively be detected for GABARAP but not the other family members in both applications. Besides its good selectivity, particularly anti-GABARAP (8H5) antibody revealed a very good signal-to-noise ratio of the GABARAP-related ELISA signal, assuming a considerably high affinity of anti-GABARAP (8H5) antibody to its given antigen, a promising feature for immunostaining applications with fixed cells.

### Anti-GABARAP (8H5) antibody recognizes the GABARAP amino-terminal region

**Figure 2:**
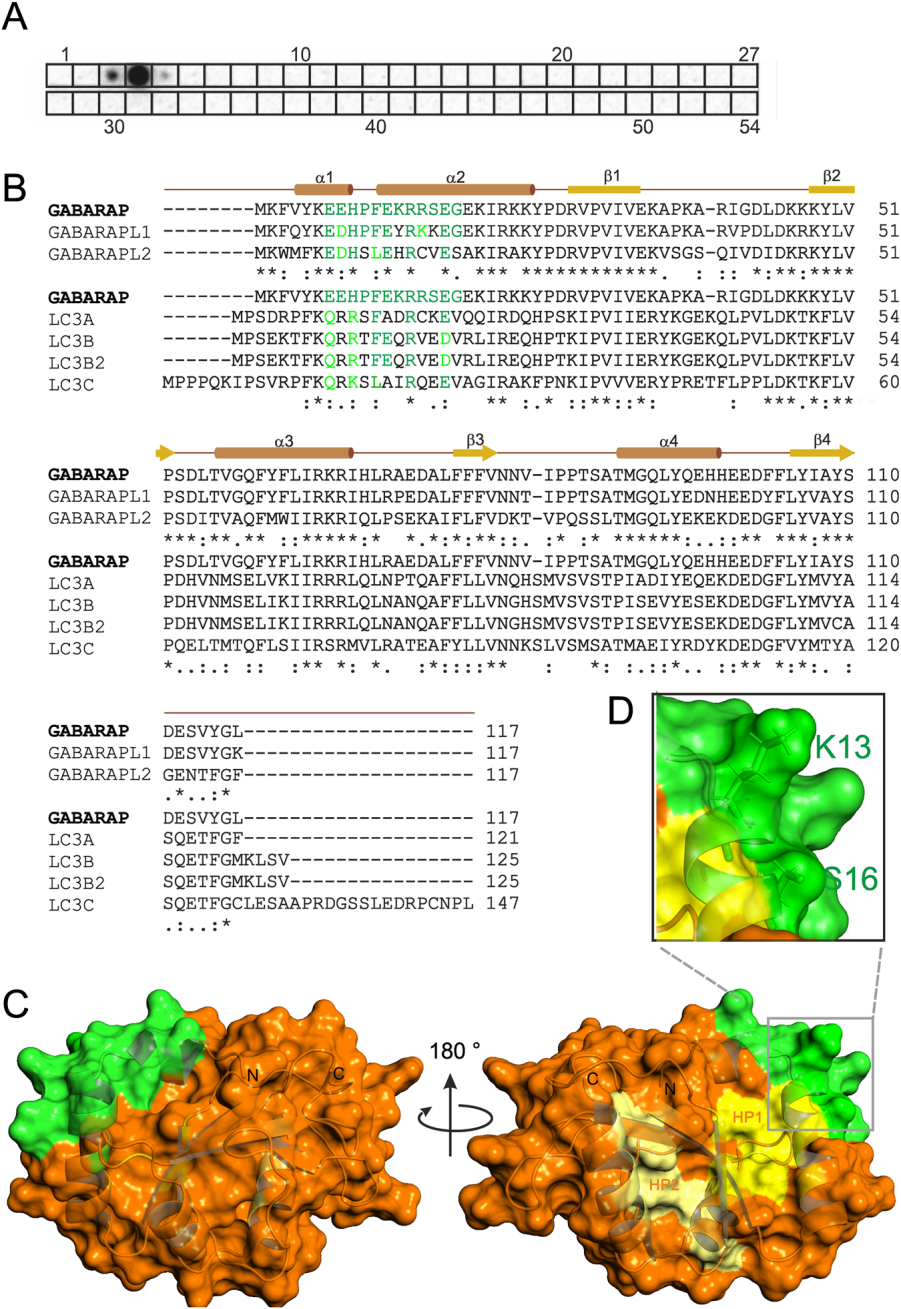
Anti-GABARAP (8H5) antibody binds an amino-terminally located epitope within GABARAP. (**A**) Peptide scanning was performed using a cellulose membrane loaded with 12mer peptides representing the complete sequence of human GABARAP and overlapping by 10 amino acids, respectively. Bound anti-GABARAP (8H5) antibody was detected using HRP-coupled mouse anti-rat IgG2a secondary antibody. Shown is one representative result. For reason of clarity peptide spot positions are boxed. Figure S3C includes source blots and membranes. (**B**) Sequence alignment of the GABARAPs (top) and of GABARAP with the LC3 family members (bottom) using Clustal Ω^42^. Regular secondary structure elements are depicted above the GABARAP sequence. The identified anti-GABARAP (8H5) antibody epitope is colored green within the GABARAP sequence. Identical residues in the sequences of the homologs listed are depicted in green, while highly similar residues are highlighted in light green. (**C**) Illustration of the anti-GABARAP (8H5) antibody epitope on the GABARAP structure (PDB ID: 1KOT) shown as combined ribbon and surface diagram using PyMOL (v1.860). Residues forming the epitope are shown in green, those forming the hydrophobic pockets HP1 and HP2 are colored dark and light yellow, respectively. In (**D**) the side chains of K13 and S16 are highlighted to illustrate their surface exposure and availability for anti-GABARAP (8H5) antibody binding.

To identify the binding epitope of anti-GABARAP (8H5) antibody which was raised against full-length GABARAP, an array-based oligo-peptide scanning was performed. In total 54 dodecapeptides (12mers) with a peptide-peptide overlap of 10 amino acids, which represent the complete sequence of hGABARAP, were spotted on a cellulose membrane. Anti-GABARAP (8H5) antibody showed a strong signal with the fourth 12mer and weak signals with the adjacent 12mers three and five, suggesting that the epitope recognized by anti-GABARAP (8H5) antibody is formed by the GABARAP residues ranging from position 7 to 18 (Fig. 2A). The alignment of GABARAP, -L1, and -L2 shows that the epitope forming region is highly similar between GABARAP and GABARAPL1, with 10 out of 12 positions being conserved (GABARAPL2: 7/12). In contrast, this region is much more variable when GABARAP is compared with the LC3s (Fig. 2B). Structurally, the identified epitope overlaps with the second half of helix α1 and first half of helix α2 (Fig. 2C), which N-terminally extend the ubiquitin-like fold that is highly conserved between all hATG8s. With K13 and S16 both non-conserved epitope residues between GABARAP and GABARAPL1 (and also -L2) expose their sidechains for antibody binding (Fig. 2D), suggesting that K13 and S16 define binding specificity of anti-GABARAP (8H5) antibody.

### Anti-GABARAP (8H5) antibody specifically stains YFP-GABARAP but not YFP-GABARAPL1 and CFP-GABARAPL2 in HAP1 GABARAP-KO cells by IF staining

**Figure 3:**
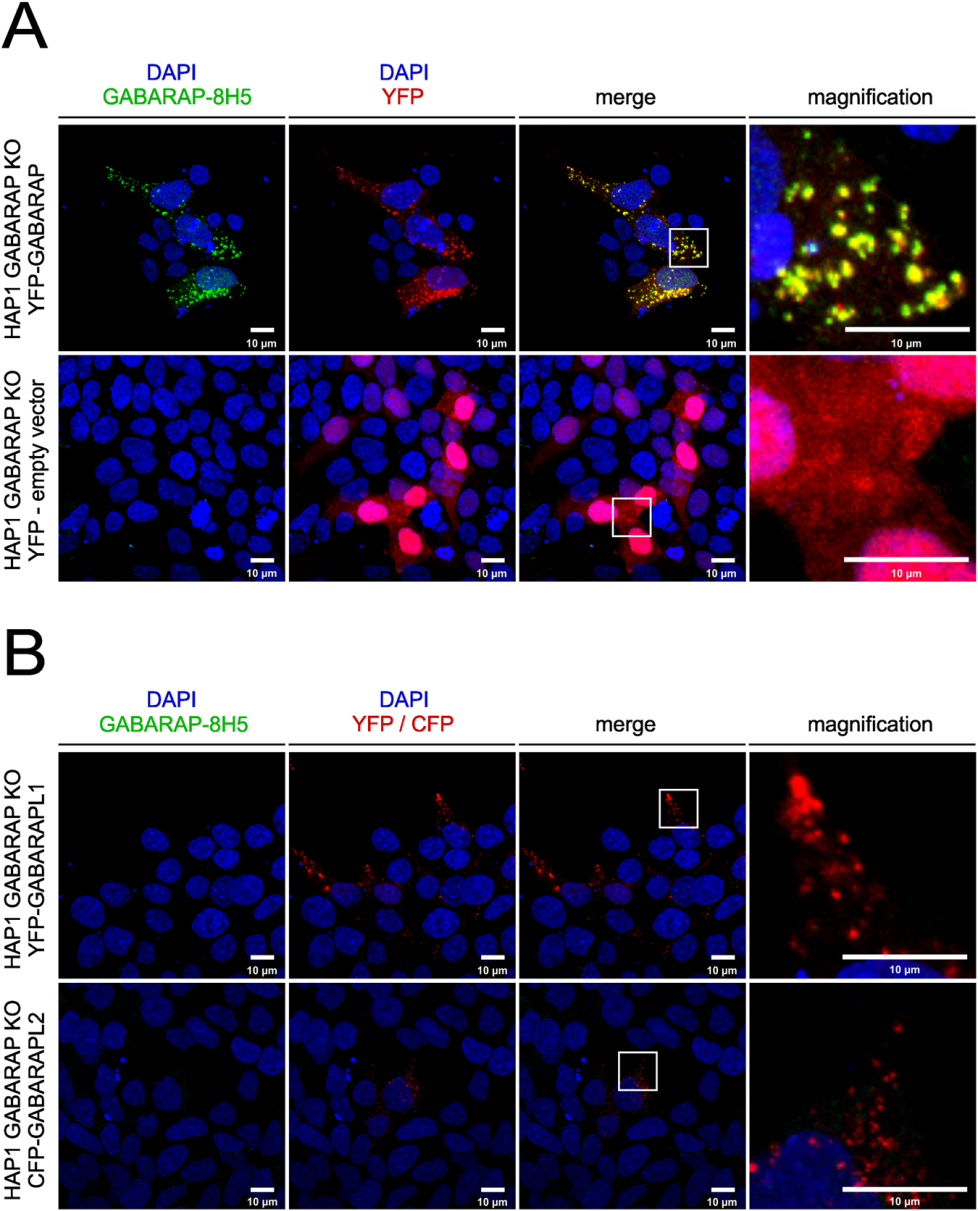
Anti-GABARAP (8H5) antibody detects YFP-GABARAP, but not YFP-GABARAPL1 and CFP-GABARAPL2 in immunofluorescence. HAP1 GABARAP KO cells were transfected with YFP-GABARAP and YFP-empty vector (**A**) or YFP-GABARAPL1 and CFP-GABARAPL2, respectively (**B**). After 48 h cells were incubated for 3 h with 100 nM Bafilomycin A1 (BafA1) in growth factor depleted medium (w/o FCS). Fixed cells (4**%** PFA) were immunolabeled with anti-GABARAP (8H5) antibody. Colocalization of GABARAP (green) and YFP (red) is indicated by yellow puncta. Nuclei were counterstained with DAPI.

In order to study if anti-GABARAP (8H5) antibody successfully stains GABARAP in IF application in fixed cells, YFP (yellow fluorescent protein)-GABARAP or YFP alone were transiently overexpressed in HAP1 GABARAP knockout (KO) cells. To do so, cells were incubated in growth factor-depleted medium in the presence of Bafilomycin A1 (BafA1). This macrolide antibiotic drug inhibits the vacuolar type H^+^-ATPase (adenosine triphosphatase) and thereby blocks the autophagic flux by preventing the fusion between autophagosomes and lysosomes^20,21^. Under these conditions, the lipidated form of YFP-GABARAP is well known to be associated to the inner and outer lipid bilayer of mature autophagosomes, which under BafA1 treatment accumulates within the cytoplasm, and can be visualized easily as bright puncta in confocal imaging applications^2,12^. As expected, in the channel used for YFP detection, punctate YFP-GABARAP accumulations and a faint cytoplasmic stain were observed within the transfected HAP1 GABARAP KO cells (Fig. 3A, top, red). Likewise, immunostaining of these cells with anti-GABARAP (8H5) antibody and a secondary antibody conjugated with Cy5 produced many puncta (green) that showed a near to perfect colocalization with the YFP-GABARAP signals obtained (merge). In contrast, transfection with the YFP control vector produced fluorescence signals in the nuclear and cytoplasmic region solely in the channel for YFP detection (Fig. 3A, bottom, red), confirming that anti-GABARAP (8H5) antibody was reacting with the GABARAP part of the YFP-GABARAP fusion protein.

To further analyze the specificity of anti-GABARAP (8H5) antibody, plasmids encoding YFP-GABARAPL1 and CFP (cyan fluorescent protein)-GABARAPL2 were transfected in HAP1 GABARAP KO cells, respectively. Transfected cells showed fluorescence exclusively in the channels for YFP or CFP detection, but not after staining with anti-GABARAP (8H5) antibody (Fig. 3B), indicating that the antibody does not cross-react with its closest relatives, GABARAPL1 and GABARAPL2, even under overexpressing conditions.

### Anti-GABARAP (8H5) antibody detects endogenous GABARAP, but not its close relatives -L1 and -L2 in IF

**Figure 4:**
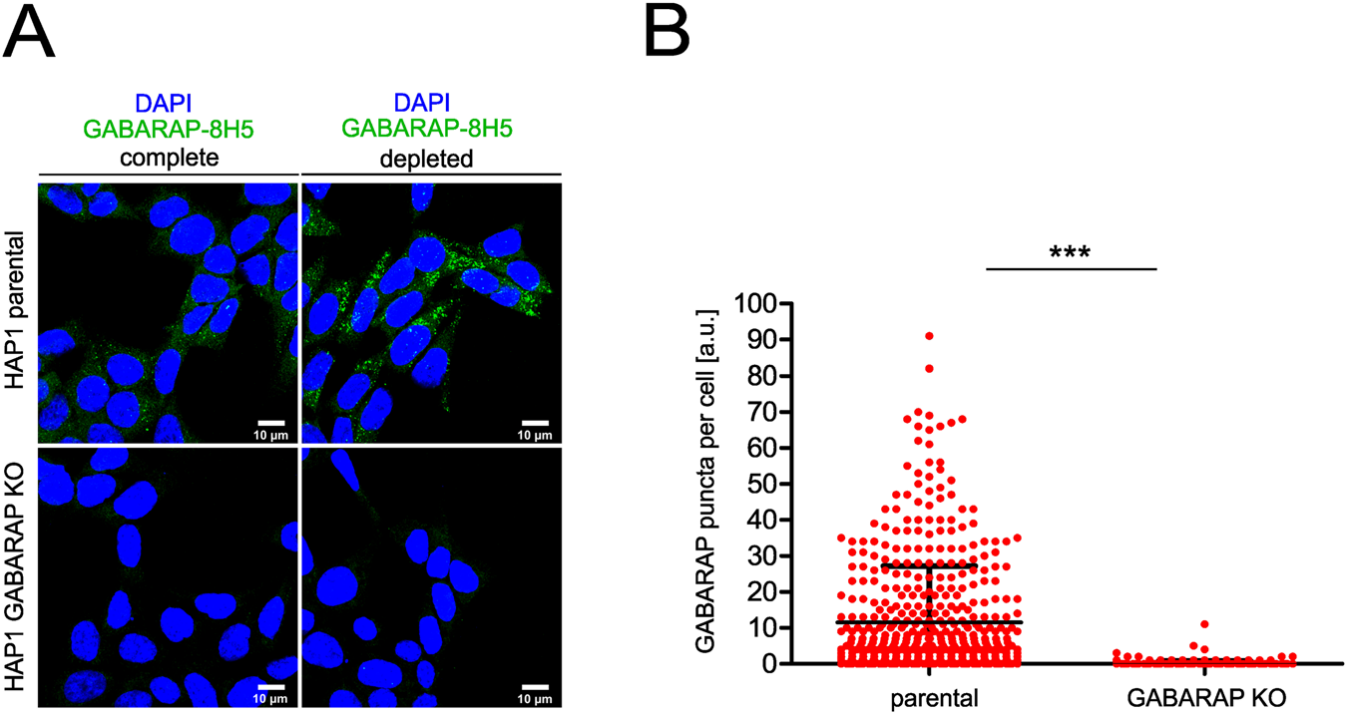
Anti-GABARAP (8H5) antibody specifically detects endogenous GABARAP in immunofluorescence. (**A**) HAP1 parental and GABARAP KO cells were incubated for 4 h in complete growth medium (IMDM + 10% FCS) and growth factor depleted medium (IMDM w/o FCS) with 100 nM Bafilomycin A1 (BafA1), respectively. Fixed cells were immunolabeled with anti-GABARAP (8H5) antibody. Nuclei were counterstained with DAPI. (**B**) Counted GABARAP puncta per cell in HAP1 cells. HAP1 cells grown under growth factor deprivation and BafA1 incubation (**A**) were analyzed. GABARAP puncta per cell from five individual experiments were measured in parental (n = 513) and GABARAP KO (n = 460) cells by Fiji analysis tool in combination with an in-house developed macro for puncta analysis. Error bars represent mean ± SD. Significance was determined using unpaired one-tailed t-test with Welch’s correction (***p < 0.0001).

To further investigate if anti-GABARAP (8H5) antibody specifically stains endogenous GABARAP, HAP1 parental control cells expressing all ATG8s at endogenous levels, and HAP1 GABARAP KO cells, lacking GABARAP but expressing GABARAPL1 and -L2 were stained. Before fixation and immunostaining with anti-GABARAP (8H5) antibody, HAP1 cells were cultured in complete growth medium (IMDM + 10% FCS) without BafA1 or under growth factor deprivation (IMDM w/o FCS) with BafA1 to accumulate autophagosomes in the cells. As expected, we obtained a prominent cytoplasmic stain accompanied with some punctate structures when staining parental HAP1 cells with anti-GABARAP (8H5) antibody under basal autophagic conditions (Fig. 4A, top. left). After growth factor depletion and BafA1 treatment, anti-GABARAP (8H5) antibody staining revealed an accumulation of many bright GABARAP-positive puncta (Fig. 4A, top, right). Only a weak background stain was obtained with anti-GABARAP (8H5) antibody when cells lacking GABARAP but expressing all the other ATG8s at endogenous levels were used (immunoblot (IB) results of GABARAPL1, -L2 in HAP1 KO cells: please refer to Supplementary Fig. S1). A quantitative analysis of in total 513 parental cells and 460 GABARAP KO cells from 5 separate stains imaged under growth factor depletion and BafA1 treatment is given in Fig. 4B, confirming the selectivity of anti-GABARAP (8H5) antibody for GABARAP at high significance (p < 0.0001).

In parallel, we also tested three commercially available anti-GABARAP antibodies. Each of them selectively detects GABARAP in immunoblotting upon SDS-PAGE (see Supplementary Fig. S1B). Interestingly, the same antibodies showed pronounced unspecific staining in GABARAP KO cells during IF in our hands (see Supplementary Fig. S2), justifying the need for an in-depth application-based antibody characterization, and impressively illustrating that specificity features of antibodies should not be transferred between different applications without separate experimental proof. In addition, we examined the performance of the anti-GABARAP (8H5) antibody in tissue sections in a preliminary manner. Therefore, two brain regions (motorcortex, hippocampus) derived from mice with a GABARAP^+/+^ background were applied to IF. In contrast to the negative control clear intracellular staining of different intensities could be obtained only in presence of anti-GABARAP (8H5) antibody (Supplementary Fig. S4). Thus, this anti-GABARAP antibody might also be applicable for IF-labelling of tissue sections.

### Anti-GABARAP (8H5) antibody distinguishes LC3B^+^/GABARAP^+^ from LC3B^+^/GABARAP^-^ vesicular structures in two-color IF

**Figure 5:**
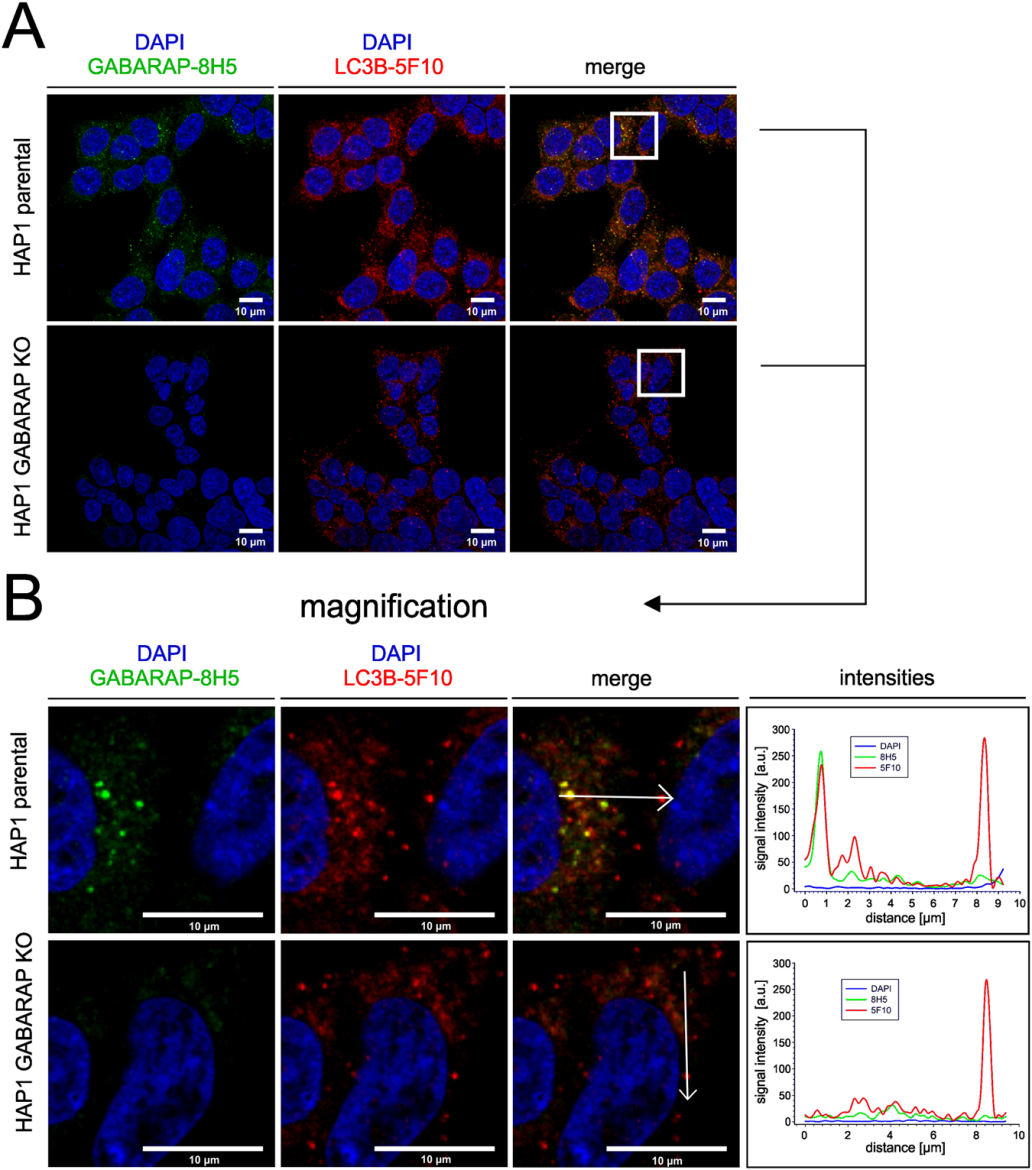
Anti-GABARAP (8H5) antibody detects endogenous GABARAP within some but not all LC3B^+^ structures. (**A**) HAP1 parental and GABARAP KO cells were incubated for 3 h with 100 nM BafA1 in growth factor depleted medium. HAP1 cells were fixed with 4**%** PFA and immunolabeled with anti-GABARAP (8H5) and anti-LC3B (5F10) antibodies. Colocalization of LC3B (red) and GABARAP (green) is indicated by yellow puncta. Nuclei were counterstained with DAPI. (**B**) Intensity profiles of line segments (approx. 10 µm) drawn across the z-section shown for each channel. Overlap of anti-LC3B (5F10) (red) and anti-GABARAP (8H5) (green) antibodies’ fluorescence intensity profiles (arbitrary units, a.u.) indicates colocalization of GABARAP with the autophagosomal marker LC3B.

Finally, we tested anti-GABARAP (8H5) antibody performance in two-color IF applications and chose LC3B as the second hATG8 to be targeted (to our knowledge, GABARAPL1 and -L2 trustworthy antibody-based analysis tools for IF are yet still lacking). We used anti-LC3B mAb clone 5F10 (anti-LC3B (5F10)), because this antibody reliably detects LC3B only and not the other LC3 family members^22^. LC3B is a common marker for autophagosomal structures^23^, and consequently, anti-LC3B (5F10) antibody (Fig. 5A in red) stained a multitude of autophagosomes coated with endogenous LC3B under growth factor depletion and BafA1 treatment, regardless whether HAP1 parental or HAP1 GABARAP KO cells were imaged. Overall, anti-GABARAP (8H5) antibody (green) stained a smaller number of punctate structures compared to anti-LC3B (5F10) antibody in the parental cell line. Clearly, magnification and intensity plot (Fig. 5B) revealed that several of the imaged bright puncta are positive for LC3B but are negative for GABARAP under the conditions used. A comparable staining pattern, but with less GABARAP positive puncta after starvation, could also be observed in human embryonic kidney 293 (HEK293) cells (Supplementary Fig. S5). This is consistent with the common view that LC3s and GABARAPs have concerted but also can exhibit distinct, non-overlapping intracellular localizations, which is accompanied with at least partial functional divergences^2,16,22,24,25^.

## Discussion

Antibodies rank amongst the most widespread tools in basic life science research. Concurrently, an extensive discourse regarding antibody performance is ongoing with the task to guard against misleading conclusions drawn from experiments that included insufficiently validated antibodies^19,26^. A validated antibody must show specificity, selectivity, and reproducibility in the context of its application. But antibody validation beyond the datasheet knowledge can be tedious, is frequently underestimated, and thus is widely ignored in project scheduling. By comparing antibody-based IF stains with their respective fluorescence protein (FP)-tagged antigen location, a recent report impressively demonstrates the high error rates of inadequately validated polyclonal antibodies during IF applications under high throughput conditions^27^.

Autophagy as well as the characterization of mATG8 function, one of the key players in this process, is subject of intensive studies. Because autophagy is proven to take part in several human e.g. neurodegenerative diseases^28,29^, deciphering the common and the unique features of individual ATG8 family members would contribute to our understanding of their underlying molecular mechanisms. Antibodies specific for each single mATG8 would improve the toolbox needed to elucidate the unique ATG8s’ functions. GABARAP, -L1, and -L2 are closely related, short-sized proteins with 117 aa (or 116 aa post processing through ATG4) in length. They, *per se*, display a limited number of epitopes, and cross-reactivity with antibodies that originally had been raised against one of their relatives, have frequently been observed in the past^15^. However, some of the available anti-GABARAP, -L1, and -L2 antibodies exhibit a specific performance with denatured proteins, e.g. in western blotting (Supplementary Fig. S1). To our knowledge, no single antibody with proven specificity during IF has been described in the literature yet. The reason for this may be that the number of exposed linear epitopes, and thus the chance of targeting a unique epitope (as basis for antibody specificity), is generally higher under denaturating than under native or semi-native conditions. Conversely, discontinuous epitopes are destroyed by denaturation, but are exposed under native or semi-native conditions, e.g. as prevailing during dot blot, ELISA or after mild cross-linking used in bio-imaging.

In this study, we positively-screened almost 40 cell clones, which generate monoclonal antibodies against hGABARAP. Out of them, we established and characterized clone 8H5 in particular with the aim to validate its performance especially during IF. In sum, hybridoma supernatants containing anti-GABARAP (8H5) antibody showed high specificity for GABARAP without cross-reactivity for the other (recombinant and purified) ATG8 homologues GABARAPL1, -L2 or LC3A, -B, and -C both in dot blot (Fig. 1A) and in western blotting (Fig. 1C). In addition to its good selectivity anti-GABARAP (8H5) antibody was our best performer during ELISA regarding binding strengths (Fig. 1B). Notably, anti-GABARAP (8H5) antibody failed to detect endogenous, SDS-PAGE-separated GABARAP from mammalian cell lysates during subsequent IB in our hands. Possible reasons are the low abundance of GABARAP in cell lysates compared to the applied amount of purified recombinant protein samples in Fig. 1C and/or the denaturation of the epitope recognized by 8H5 during western blotting^30,31^. Interestingly, epitope mapping of anti-GABARAP (8H5) antibody revealed that it interacts with the α–helices 1 and 2 containing region (Fig. 2), suggesting that the existence of some α-helical secondary structure supports its efficient binding. According to the GABARAP structure a proper positioning of the non-conserved residues K13 and S16, which both are located within the binding epitope might be pivotal through acting as a GABARAP-unique platform for antibody binding. Because the complete GABARAP protein was applied as immunogen, it is likely, that under native conditions further parts of the GABARAP molecule contribute here by delivering the required scaffold for an optimal epitope presentation.

Following the latest recommendations for antibody validation^19^, we next characterized the performance of anti-GABARAP (8H5) antibody during IF staining applications in-depth. We compared the anti-GABARAP (8H5) antibody-based staining with that of FP-tagged GABARAP, GABARAPL1, and GABARAPL2 ectopically expressed in HAP1 cells lacking endogenous GABARAP (Fig. 3A,B). Next, we compared the staining pattern of cells expressing all mATG8 family members at endogenous levels with that of cells lacking GABARAP, both under fed and growth-factor depleted/BafA1-treated conditions (Fig. 4). Furthermore, cells were simultaneously stained with anti-GABARAP (8H5) antibody and an antibody specifically recognizing LC3B, which is a common and independent marker for autophagosomes (Fig. 5).

Taken together, our results demonstrate that anti-GABARAP (8H5) antibody performs with high specificity in IF experiments. To our knowledge, we hereby provide the first staining results of endogenous GABARAP with an application- and target-specific in-depth validated antibody. In our opinion, this antibody can be very valuable for future studies that are aimed to resolve unique GABARAP functions in diverse cellular processes with already proven or assumed GABARAP-participation including autophagy, protein trafficking or unconventional secretion.

Recently, fluorescence protein-tagged, peptide-based sensors that can target mATG8s in a (semi)-specific manner have been published^32,33^. These sensors are based on peptides with LC3-interacting region (LIR) properties, showing selectivity, e.g. for GABARAP/GABARAPL1- or LC3B/LC3A-positive autophagosomes^32^, or even for individual mATG8s^33^. Here, further engineering including the addition of domains for membrane recruitment or oligomerization, was necessary to achieve both selectivity and sufficient affinity of these sensors. While such sensors are very valuable for live-cell visualization of membrane-associated, lipidated mATG8 forms, and those ATG8 populations without tightly complexed intracellular LIR-containing binding partners, they likely fail to visualize both their soluble, unlipidated forms and their LIR-ligand complexed forms. We assume that anti-GABARAP (8H5) antibody can react with unlipidated GABARAP, and - because its epitope is largely separated from HP1 and HP2 - with such GABARAP molecules that are bound to an LIR-ligand. However, because its epitope includes with K13 one of the two ubiquitination sites (K13, K23) that recently have been identified for GABARAP^18^, at least the K13-ubiquitinated GABARAP fraction will very likely not be stained by anti-GABARAP (8H5) antibody. Since its epitope overlaps with the microtubule-binding domain (amino acid residues 1–22) of GABARAP^34^, further work is needed that has to clarify how anti-GABARAP (8H5) antibody performs with potentially microtubule-associated GABARAP molecules. Thus, anti-GABARAP (8H5) antibody not only discriminates GABARAP from its relatives but also opens avenues to distinguish between distinct cellular pools of GABARAP itself, a feature that can help to decipher distinct roles for the diverse variations of the same protein. Finally, because mammalian GABARAP sequences display 100% conservation, anti-GABARAP (8H5) antibody can be broadly used across mammalian species. Even within orthologous proteins from less related vertebrate, arthropode and nematode model organisms the anti-GABARAP (8H5) antibody epitope surrounding region is highly conserved (Supplementary Fig. S6). Thus, this antibody will likely be useful also in non-mammalian species. For improved results, the combination with anti-rat IgG2a subclass-specific secondary antibodies, especially for multiple labeling methods, is recommended.

## Methods

### Eukaryotic plasmids

Genes for GABARAP, GABARAPL1 and GABARAPL2 were subcloned from GST-fusion plasmids (Addgene IDs 73948, 73945 and 73518) by PCR amplification into the XhoI and BamHI sites of peYFP-C1 or  peCFP-C1(Clontech), yielding peYFP-C1/GABARAP, peYFP-C1/GABARAPL1 and peCFP-C1/GABARAPL2.

### Antibodies

The antibodies used throughout this study are listed in Supplementary Table S3.

### Recombinant protein/antigen expression and purification

Cloning, expression and purification of human GABARAP (aa 1–117), GABARAPL1 (aa 1–117), GABARAPL2 (aa 1-117), MAP1-LC3A (aa 1–121), MAP1-LC3B (aa 1–125) and MAP1-LC3C (aa 1-147) was performed as previously described^35,36^.

### Antibody generation

Purified full length GABARAP protein N-terminally fused to glutathione-S-transferase (GST) was used as antigen for immunization. Approximately 50 µg of antigen dissolved in phosphate-buffered saline (PBS) was emulsified in an equal volume of incomplete Freund’s adjuvant (Sigma-Aldrich, Germany) and 5 nmol CpG2006 (TIB MOLBIOL, Germany) and injected both intraperitoneally (ip) and subcutaneously (sc) into Lou/C rats. After 6 weeks, the animals received a booster injection (sc and ip) with 50 µg of antigen without Freund’s adjuvant. Fusion of the myeloma cell line P3X63-Ag8.653^37^ with immune spleen cells was performed according to the standard procedure described by Köhler and Milstein^38^. After fusion, the cells were cultured in 96-well cluster plates in standard medium supplemented with 20% fetal calf serum (FCS), 2% HCS (Capricorn Scientific, USA), and aminopterin (Life Technologies, Germany).

Hybridoma supernatants were tested in a solid-phase enzyme-linked immunoassay (ELISA). Plates were coated overnight with mouse-anti GST antibody (5 µg/ml) and after blocking with 2% FCS, GST-GABARAP fusion protein was added at 0.7 µg/ml for 60 min. Irrelevant GST-fusion protein served as negative control. Hybridoma supernatants (1:10 diluted) were added and GABARAP-bound antibodies were detected with a mixture of horseradish peroxidase (HRP)-coupled mouse monoclonal antibodies against rat IgG heavy chains. The secondary antibodies were visualized with 3,3′,5,5′-tetramethylbenzidine (TMB) substrate (Thermo Scientific, Germany) by measuring the absorbance at 650 nm with a microplate reader (Tecan, Switzerland). The IgG subclass was determined by ELISA with mouse anti-rat light chain antibodies as capture and HRP-coupled mouse anti-rat IgG subclass-specific antibodies for detection. The hybridoma cells of clone 8H5 (IgG2a/k) were stably subcloned twice by limiting dilution.

### Dot blot analysis

Purified ATG8 family proteins (GABARAP, GABARAPL1, GABARAPL2, LC3A, LC3B and LC3C) were adjusted to a final concentration of 10 µM, respectively. 1 µl of each solution was transferred to a cellulose nitrate membrane considering an adequate spacing between all spots. After 5 min, membranes were blocked with tris-buffered saline-tween (TBS-T) (TBS, 0.1% Tween 20) including 5% nonfat-dried milk powder (AppliChem) for 30 min at room temperature (RT). The membrane was subsequently incubated with the primary antibody containing hybridoma supernatant (rat anti-GABARAP clone number 8H5) diluted in blocking buffer (1:1). Incubation was performed overnight at 4 °C. After three washing steps with TBS-T, HRP-coupled secondary antibody (mouse anti-rat-IgG2a-HRP; 1:1000) was applied for 1 h at RT. After three further washing steps with TBS-T, the membrane was incubated with the HRP substrate SuperSignal West Pico Chemiluminescent Substrate (Thermo Scientific, Germany) for 5 min at RT. Final quantification of immunosignal was performed using a chemiluminescence detection system (ChemiDoc, Bio-Rad, Hercules, CA, USA).

### ELISA (for assessing binding specificity of selected hybridoma supernatants)

96 well Nunc-Immuno MicroWell Polysorp plates (Thermo Scientific, Germany) were coated with 700 ng of one GABARAP family protein per well and incubated for 1 h at RT. Wells were subsequently blocked for 30 min at RT using TBS-T including 5% nonfat-dried milk powder (AppliChem, Germany). Hybridoma supernatant with primary antibody (rat anti-GABARAP clone 8H5) was diluted in blocking buffer (1:10) and was further transferred to 7 wells, respectively. Each of these wells contained a distinct protein of the GABARAP family. Incubation was performed for 1 h at RT. After three washing steps using TBS-T, a suitable HRP coupled secondary antibody (goat anti-rat IgG-HRP) was diluted (1:1000) and subsequently transferred to the wells. After 1 h of incubation at RT, each well was washed three times with TBS-T. The HRP substrate and TMB (Sigma-Aldrich, T5525, Germany) was finally applied to all wells according to manufacturer’s instruction. Immunosignal was quantified at 450 nm (Fluostar Optima, BMG Labtechnologies GmbH, Germany).

### Polyacrylamide gel electrophoresis and western blot analysis

Purified ATG8 family proteins (GABARAP, GABARAPL1, GABARAPL2, LC3A, LC3B and LC3C) were denatured at 95 °C for 5 min in SDS loading buffer (10% (v/v) glycerol, 2% (w/v) SDS, 2% (v/v) 2-mercaptoethanol, 50 mM Tris, pH 6.8, 0.75 g/l bromphenol blue). ATG8 family proteins (1 µg respectively) were then applied to a 12% SDS-PAGE and separated at 40 mA for 45 min. Gel staining was performed with coomassie staining solution (25% isopropanol, 10% acidic acid, 0.5 g/l coomassie brilliant blue R-250) and the destaining was done in hot water.

For western blot analysis, proteins were transferred to a PVDF membrane (pore size 0.2 µm) without preceding gel staining. Protein transfer was performed in a semi dry system at 25 V for 60 min. The membrane was subsequently washed in TBS-T and then blocked in TBS-T including 2.5% milk powder for 30 min. The blot was incubated with primary antibody containing hybridoma supernatant (rat anti-GABARAP (8H5) antibody) diluted in TBS-T (1:4) over night at 4 °C. After washing (3 times for 5 min, respectively) in TBS-T, the membrane was incubated with HRP-conjugated secondary antibody (goat anti-rat-HRP, Jackson 112–035–068, diluted 1:2000 in TBS-T) at RT for 1 h. The blot was washed (3 times for 5 min, respectively) in TBS-T and immunoreactivity was finally quantified as described for dot blot analysis.

### Epitope mapping

Peptide scanning was performed using a cellulose membrane (Intavis AG, Germany) loaded with 12mer peptides representing the complete sequence of hGABARAP and overlapping by 10 amino acids, respectively. Every peptide was spotted on the membrane twice. Epitope mapping was done by washing the membrane with TBS-T, followed by a blocking step using TBS-T/5% nonfat-dried milk powder. After another washing step with TBS-T, primary antibody (rat anti-GABARAP (8H5) antibody hybridoma supernatant) was diluted with TBS-T (1:5) and applied over night at 4 °C. After 3 washing steps using TBS-T, HRP-coupled mouse anti-rat IgG2a antibody was diluted with TBS-T (1:2000) and applied for 2 h at RT. After another 3 washing steps with TBS-T, signal detection was performed by incubation with SuperSignal West Pico Chemiluminescent Substrate (Thermo Scientific, Germany) for 5 min at RT. Immunosignal was quantified using a chemiluminescence detection system (ChemiDoc, Bio-Rad, Hercules, CA, USA).

### Cell culture and transfection

P3X63-Ag8.653 cells were cultured at 37 °C in a humidified 5% CO_2_ incubator in standard medium RPMI 1640 (Sigma-Aldrich, Germany) supplemented with 1% glutamine, 1% non-essential amino acids, 1% sodium pyruvate, 1% penicillin/streptomycin (Sigma-Aldrich, Germany), and 2.5% FCS (Capricorn).

HAP1 cells are adherent human fibroblast-like cells with a near-haploid karyotype that have been derived from the male chronic myelogenous leukemia (CML) cell line KBM-7^39^. HAP1 parental (C631) and HAP1 GABARAP KO (HZGHC003054c004) cells were purchased from Horizon Discovery, UK. HAP1 cells were cultured at 37 °C in a humidified 5% CO_2_ incubator in growth medium Iscove’s Modified Dulbecco’s Medium (IMDM – Gibco, Thermofisher Scientific, Germany) supplemented with 1% penicillin/streptomycin (Sigma-Aldrich, Germany), and 10% FCS (Sigma-Aldrich, Germany).

For transient transfection with peYFP-N1 empty vector (Clontech, USA), peYFP-GABARAP, peYFP-GABARAPL1, and peCFP-GABARAPL2 constructs, 1 × 10^5^ HAP1 cells were seeded on a poly D-Lysine coated bottom dish (MatTek Corporation, MA, USA) and incubated in IMDM / 10% FCS, respectively. The next day transfection with 1.5 µg total DNA using Turbofectin 8.0 (OriGene, USA) was performed according to manufacturer’s instructions.

### Immunofluorescence

HAP1 cells (3 × 10^5^) were seeded on a poly D-Lysine coated glass bottom dish (MatTek Corporation, MA, USA) and incubated in IMDM / 10% FCS. The next day, IMDM was removed and HAP1 cells were incubated for 3–4 h in IMDM medium with 10% FCS only or IMDM without 10% FCS including 100 nM Bafilomycin A1 (Sigma-Aldrich, Germany). Fixation with 4% (w/v) paraformaldehyde in PBS at RT for 10 min was followed by a washing step using PBS and addition of 0.2% TritonX-100 in PBS for 30 min at RT to permeabilize the cell membranes. After three washing steps with PBS, surfaces were blocked with 1% bovine serum albumin (BSA, Sigma-Aldrich, Germany) at RT for 60 min or overnight at 4–8 °C. Immunostaining was performed by addition of 1 mL undiluted hybridoma supernatant including anti-GABARAP (8H5) antibody and 1 µg/mL mouse monoclonal anti-LC3B (5F10) antibody and incubation for 60 min at RT under smooth shaking. Cells were washed three times for 5 min with PBS followed by incubation of an appropriate 1:250 diluted fluorescent labelled secondary antibody (goat anti-rat Alexa Fluor 488 for 8H5; goat anti-mouse Alexa Fluor 647 for mAb LC3B) for 60 min at RT in the dark, followed by two washing steps for 5 min with PBS. YFP, YFP-GABARAP, and YFP-GABARAPL1 transfected HAP1 cells were fixed 2 to 3 days after transfection. Fixing and staining procedure was done as described above, and a donkey anti-rat Cy5 secondary Ab (1:250) was used in combination with anti-GABARAP (8H5) antibody.

### Image acquisition

Images were acquired using ZEN black 2009 on a LSM 710 confocal laser scanning system (Carl Zeiss MicroImaging Inc., Germany) with a Plan-Apochromat 63 × /1.40 Oil DIC M27 objective. The cell nuclei (DAPI) were measured in the 405 nm channel (MBS -405/760+). GABARAP puncta were detected in the 488 nm channel (MBS 488/543/633) and LC3B puncta in the 633 nm channel (MBS 488/543/633), respectively. YFP was detected in channel 514 nm (MBS 458/514) and Cy5 in channel 633 nm (MBS 488/543/633).

### Image evaluation

Image analysis was done using ImageJ / Fiji^40,41^. For quantitative and unbiased evaluation of anti-GABARAP (8H5) antibody a macro has been written and applied to images of parental and GABARAP KO cells. Within the macro a maximum intensity projection and a defined threshold (70/255) was used. After thresholding the images, a watershed algorithm was applied and puncta with a size >3 pixel were counted using the *Analyze Particles* tool of ImageJ. The puncta were analyzed in single cells using manually annotated regions of interest (ROIs) within the images. For better visibility in the print version, maximum intensity projections and an adjustment of brightness (20), contrast (40) and intensity (60) was applied equally for every figure using CorelDRAW 2017. For data analysis, GraphPad Prism Version 5.00 was used.

## Electronic supplementary material

The highly GABARAP specific rat monoclonal antibody 8H5 visualizes GABARAP in immunofluorescence imaging at endogenous levels

## Data Availability

No datasets were generated or analyzed during the current study.
