# Author Correction: The highly GABARAP specific rat monoclonal antibody 8H5 visualizes GABARAP in immunofluorescence imaging at endogenous levels

**DOI:** 10.1038/s41598-019-55814-3

**Published:** 2019-12-11

**Authors:** Indra M. Simons, Jeannine Mohrlüder, Regina Feederle, Elisabeth Kremmer, Thomas Zobel, Jochen Dobner, Nicole Bleffert, Silke Hoffmann, Dieter Willbold

**Affiliations:** 1 Institut für Physikalische Biologie, Heinrich-Heine-Universität, 40225 Düsseldorf, Germany; 2 Institute of Complex Systems, Structural Biochemistry (ICS-6), Forschungszentrum Jülich, 52425 Jülich, Germany; 3 Institute for Diabetes and Obesity, Monoclonal Antibody Core Facility, 85764 Neuherberg, Germany; 4 Institute for Molecular Immunology, Helmholtz Center Munich, German Research Center for Environmental Health, 85764 Neuherberg, Germany; 5 Department of Biology, Center for Advanced Imaging (CAi), Heinrich-Heine-Universität, 40225 Düsseldorf, Germany

Correction to: *Scientific Report* 10.1038/s41598-018-36717-1, published online 24 January 2019

This Article contains typographical errors in the Acknowledgements section.

“This study was funded by the Deutsche Forschungsgemeinschaft (DFG, German Research Foundation) – Projektnummer 190586431 – SFB 974, project B02 (D.W., I.M.S.), and project WE5343/1–1 (T.Z.), and by a grant from the Jürgen Manchot Foundation, Molecules of Infection Graduate School (MOI III) (D.W. and J.D.).”

should read:

“This study was funded by the Deutsche Forschungsgemeinschaft (DFG, German Research Foundation) – Projektnummer 267205415 – SFB 1208, project B02 (D.W., I.M.S.), and project WE5343/1–1 (T.Z.), and by a grant from the Jürgen Manchot Foundation, Molecules of Infection Graduate School (MOI III) (D.W. and J.D.).”

