# Supplementary material for: The highly GABARAP specific rat monoclonal antibody 8H5 visualizes GABARAP in immunofluorescence imaging at endogenous levels
Source: Sci Rep. 2019 Jan 24;9:526. doi: 10.1038/s41598-018-36717-1 (PMC6346085; doi:10.1038/s41598-018-36717-1)
Supplement: Supplementary file 1 — The highly GABARAP specific rat monoclonal antibody 8H5 visualizes GABARAP in immunofluorescence imaging at endogenous levels [file 41598_2018_36717_MOESM1_ESM.docx]

# Supplementary Info

**The highly GABARAP specific rat monoclonal antibody 8H5 visualizes GABARAP in immunofluorescence imaging at endogenous levels**

Indra M. Simons^1^, Jeannine Mohrlüder^2^, Regina Feederle^3^, Elisabeth Kremmer^4^, Thomas Zobel^5^, Jochen Dobner^1^, Nicole Bleffert^1^, Silke Hoffmann^2^, Dieter Willbold^1,2^

^1^Institut für Physikalische Biologie, Heinrich-Heine-Universität, 40225 Düsseldorf, Germany

^2^Institute of Complex Systems, Structural Biochemistry (ICS-6), Forschungszentrum Jülich, 52425 Jülich, Germany

^3^Institute for Diabetes and Obesity, Monoclonal Antibody Core Facility ^4^Institute for Molecular Immunology Helmholtz Center Munich, German Research Center for Environmental Health, 85764 Neuherberg, Germany

^5^Department of Biology, Center for Advanced Imaging (CAi), Heinrich-Heine-Universität, 40225 Düsseldorf, Germany

Supplementary Methods are related to Figure S1, S2, S4 and S5

Supplementary Figure 1 is related to all Figures dealing with HAP1 cells (Fig. 3, 4, 5)

Supplementary Figure 2 is related to Figure 4

Supplementary Figure 3 is related to Figures 1A, 1C, 2A, S1A and S1B

Supplementary Figure 4

Supplementary Figure 5 is related to Figure 5

Supplementary Figure 6

Supplementary Table 1 is related to Figure S1 B

Supplementary Table 2 is related to Figure Table S1 and Supplementary Figure S1 B

Supplementary Table 3 is related to all figures

Supplementary References are related to Figure S1

## Supplementary Methods

**Western blotting of mammalian cell lysates**

For immunoblots depicted in figure S1, cells were washed once with warm PBS, dissociated with Trypsin-EDTA (Cytogen, Wetzlar, Germany) for 5 min, resuspended in medium and centrifuged for 3 min at 4 °C and 900 g. Afterwards, cell pellets were washed once with cold PBS and resuspended in lysis buffer (136 mM NaCl, 20 mM Tris-HCl, 10 % Glycerin, 2 mM EDTA, 50 mM β-Glycerophosphat, 20 mM Na-Pyrophosphat, 0.2 mM Pefablock, 5 µg/ml Aprotinin, 5 µg/ml Leupeptin, 4 mM Benzamidin, 1 mM Na_3_VO_4_ , 0.2 % SDS, pH 7.4) and frozen for 20 min at -20 °C. Subsequently, samples were thawed, sonicated thrice for 15 seconds at 100 % on ice and centrifuged for 15 min at 4 °C and 20 000 g to get rid of cell debris. Supernatants were transferred to fresh tubes and protein content measured with Bradford protein assay (Biorad, California, USA). 40 µg of whole cell protein lysates were then subjected to 12 % polyacrylamid SDS-PAGE. Proteins were then transferred onto a PVDF membrane via Semi-Dry Western Blot. Membranes were blocked with either 5 % milk in TBS-T or 5 % BSA in TBS-T for 1 h at room temperature (RT), followed by three times 20 min washing with TBS-T at RT incubation with primary antibody overnight at 4 °C and secondary HRP-coupled antibody for 1 h at RT. Blots were visualized by chemiluminescence (Western Lightning Plus-ECL, Perkin Elmer, Massachusetts, USA) and documented using the ChemiDoc system (Biorad, California, USA) or film (Amersham Hyperfilm ECL, GE, UK).

**Generation of HEK293 knockout cell lines**

HEK293 cells were nucleofected (Lonza, Basel, CH) with KO plasmids targeting *GABARAP* exon 1, *-L1* exon 2 and *-L2* exon 2 coexpressing either GFP, CFP or mCherry. KO plasmids are based on plasmid pSpCas9(BB)-2A-GFP (PX458), which was a gift from Feng Zhang (Addgene plasmid # 48138)^1^. Clonal lines were created by growth of single cell sorted FP positive cells via fluorescence activated cell sorting (FACS). Genomic mutations were validated via Sanger sequencing of PCR amplificates of the sgRNA target region +/- 200 bp (table S1) and analysed by TIDE and Crisp-ID^2,3^, as listed in table S2.

**Ethics statement**

All animal experiments were performed in accordance with the German Law on the protection of animals (TierSchG §§ 7-9) and with permit from the local ethic committee (Landesamt für Natur, Umwelt und Verbraucherschutz Nordrhein-Westfalen (LANUV), North Rhine-Westphalia, Germany; AZ 84-02.04.2015.A106 and AZ 84-02.04.2014.A423).

**Immunofluorescent analysis of mouse brain slices**

Endogenous GABARAP in tissue sections was assessed by immunofluorescence analysis using 20 µm thick, sagittal sections of 20 weeks old mice divided by a cryostat (CM3050 S, Leica Biosystems Nussloch GmbH, Wetzlar, Germany). Formalin embedded brain sections were fixed for 10 min with 4 % PFA, three times washed for 5 min with (Tris-buffered saline) TBS and treated for 10 min with 70 % formic acid for antigen retrieval, followed by another three 5 min washing steps with TBS. After permeabilization with 1 % TBS-Triton-X (TBS-T) for 30 min, brain sections were blocked with 1 % BSA in TBS over night at 4 °C. Incubation with anti-GABARAP (8H5) antibody was performed with undiluted hybridoma supernatant for 2 h at RT. After three washing steps for 5 min with TBS, sections were incubated for 1 h with a goat anti-rat IgG+IgM (H&L) Alexa Fluor 488 secondary antibody (112-545-068, Dianova, 1:250 in 1 % BSA in TBS), followed by three 5 min washing steps with TBS. In addition, the secondary antibody was applied in the absence of primary antibody, to assess the specificity of the stain, and all residual staining was considered to be non-specific. Immunofluorescent sections were counterstained by DAPI (4,6-Diamidin-2-phenylindol) (Merck, Germany) and again washed as described. Brain sections were mounted with Aqua Poly/Mount (18606, Polysciences, Inc. Warrington, Pennsylvania, USA). Images were taken with a LSM 710 confocal laser scanning system (Carl Zeiss MicroImaging Inc., Germany) equipped with a EC Plan-Neofluar 20x/0.50 M27a or a Plan-Apochromat 63x/1.40 Oil DIC M27 objective.

## Immunofluorescence

HEK293 cells (3 x 10^5^) were seeded on a fibronectin (Sigma-Aldrich, Germany) coated glass bottom dish (ibidi, Germany) and incubated in DMEM supplemented with 10 % FCS for 24 hours. The next day, DMEM was removed and HEK293 cells were incubated for 3 h – 4 h in DMEM medium without 10 % FCS or Earle’s Balanced Salts (EBSS, Sigma-Aldrich, Germany) including 100 nM Bafilomycin A1 (Sigma-Aldrich, Germany). Fixation with 4 % (w/v) paraformaldehyde (PFA) in PBS at RT for 10 min was followed by a washing step using PBS and addition of 0.2 % TritonX-100 in PBS for 30 min at RT to permeabilize the cell membranes. After three washing steps with PBS, surfaces were blocked with 1 % bovine serum albumin (BSA, Sigma-Aldrich, Germany) overnight at 4-8 °C. Immunostaining was performed by addition of 1 mL undiluted hybridoma supernatant including anti-GABARAP (8H5) and 1 µg/mL mouse monoclonal anti-LC3B (5F10) antibody and incubation for 60 min at RT under smooth shaking. Cells were washed three times for 5 min with PBS followed by incubation of an appropriate 1:250 diluted fluorescent labelled secondary antibody (goat anti-rat Alexa Fluor 488 for 8H5; goat anti-mouse Alexa Fluor 647 for mAb LC3B) for 60 min at RT in the dark, followed by two washing steps for 5 min with PBS.

HAP1 parental and GABARAP KO cells were cultured under growth factor deprivation with 100 nM BafA1 for 3 h. Cells were fixed with 4 % (w/v) PFA in PBS at RT or with 100 % methanol for 15 min at – 20 °C, both followed by washing twice for 5 min. Cells were immunolabeled with polyclonal (pAb) GABARAP (Proteintech 1:200, abgent 1:25) or monoclonal GABARAP (Cell Signaling E1J4E, 1:200) antibodies followed by staining with Alexa Fluor 555 (pAb – 1 (Proteintech)) and with Alexa Fluor 488 (mAb (E1J4E) and pAb – 2 (abgent)).

## Supplementary Figure S1


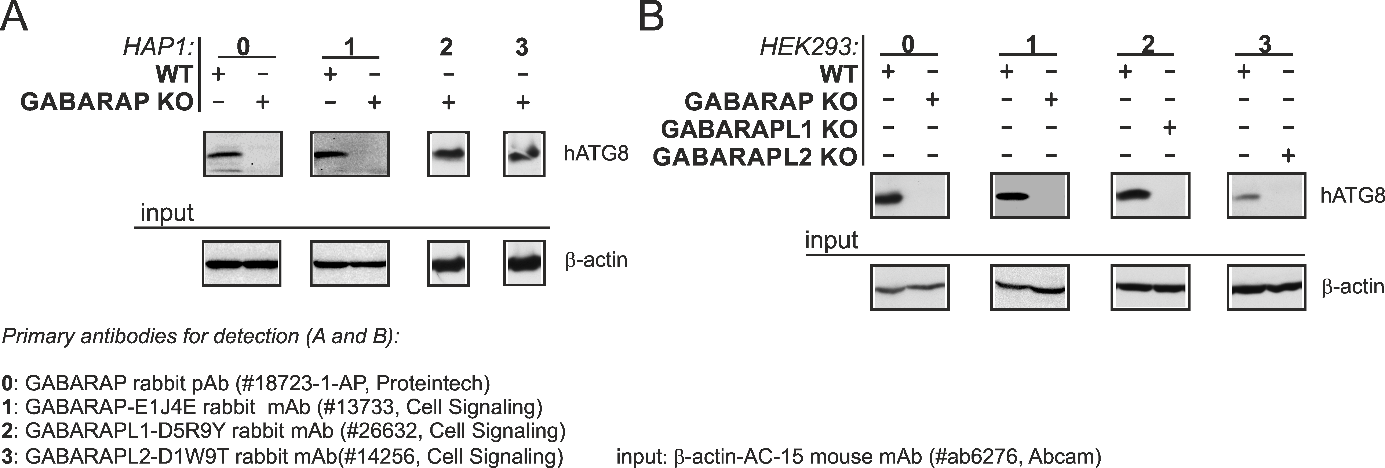


**Supplementary Figure S1. Expression analysis of GABARAP, GABARAPL1, and GABARAPL2 in HAP1 (A) and HEK293 (B) cell lysates.** (B) KO-validation of the primary antibodies used in (A) demonstrating their target specificity during immunoblotting applications. GABARAP TKO cells were created using the CRISPR/Cas9 system^1^. HEK293 KO cell lines in (B) were used to validate specificity of the antibodies used against GABARAP, GABARAP-L1 and GABARAP-L2 during immunoblotting. “hATG8” stands for the corresponding GABARAP-subfamily member, detected by the respective antibody. The corresponding full-length blots are given in Supplementary Fig. S3.

## Supplementary Figure S2


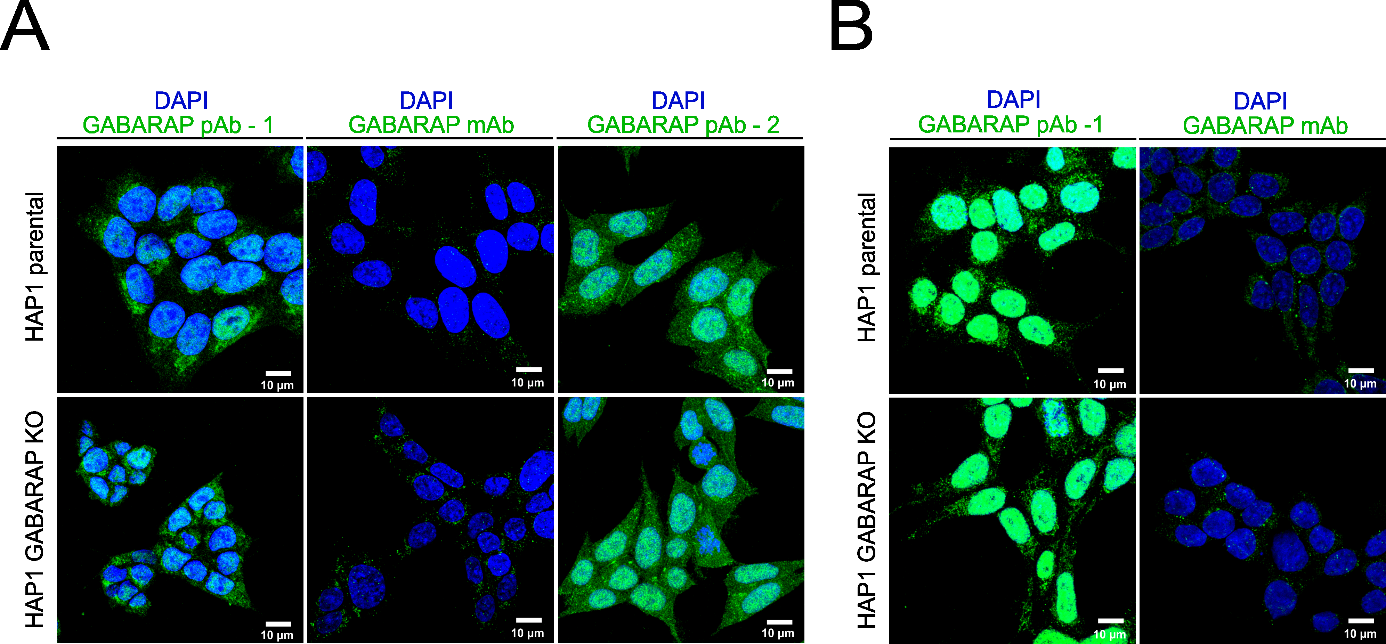


**Supplementary Figure S2.** **Commercial antibodies fail to detect endogenous GABARAP in immunofluorescence in a specific manner.** HAP1 parental and GABARAP KO cells were cultured under growth factor deprivation with 100 nM BafA1 for 3 h. Fixed cells were immunolabeled with polyclonal (pAb) GABARAP or monoclonal GABARAP antibodies. Under (A) a 4 % PFA fixation and a staining with Alexa555 (pAb – 1 (Proteintech)) and with Alexa488 (mAb (E1J4E) and pAb – 2 (abgent)) was applied. Under (B) a fixation with 4 % PFA and a staining with Alexa488 (pAb) and a methanol-based fixation protocol as recommended by the supplier for GABARAP mAb E1J4E with Alexa488 as secondary antibody was used, respectively. Nuclei were counterstained with DAPI.

## Supplementary Figure S3

**
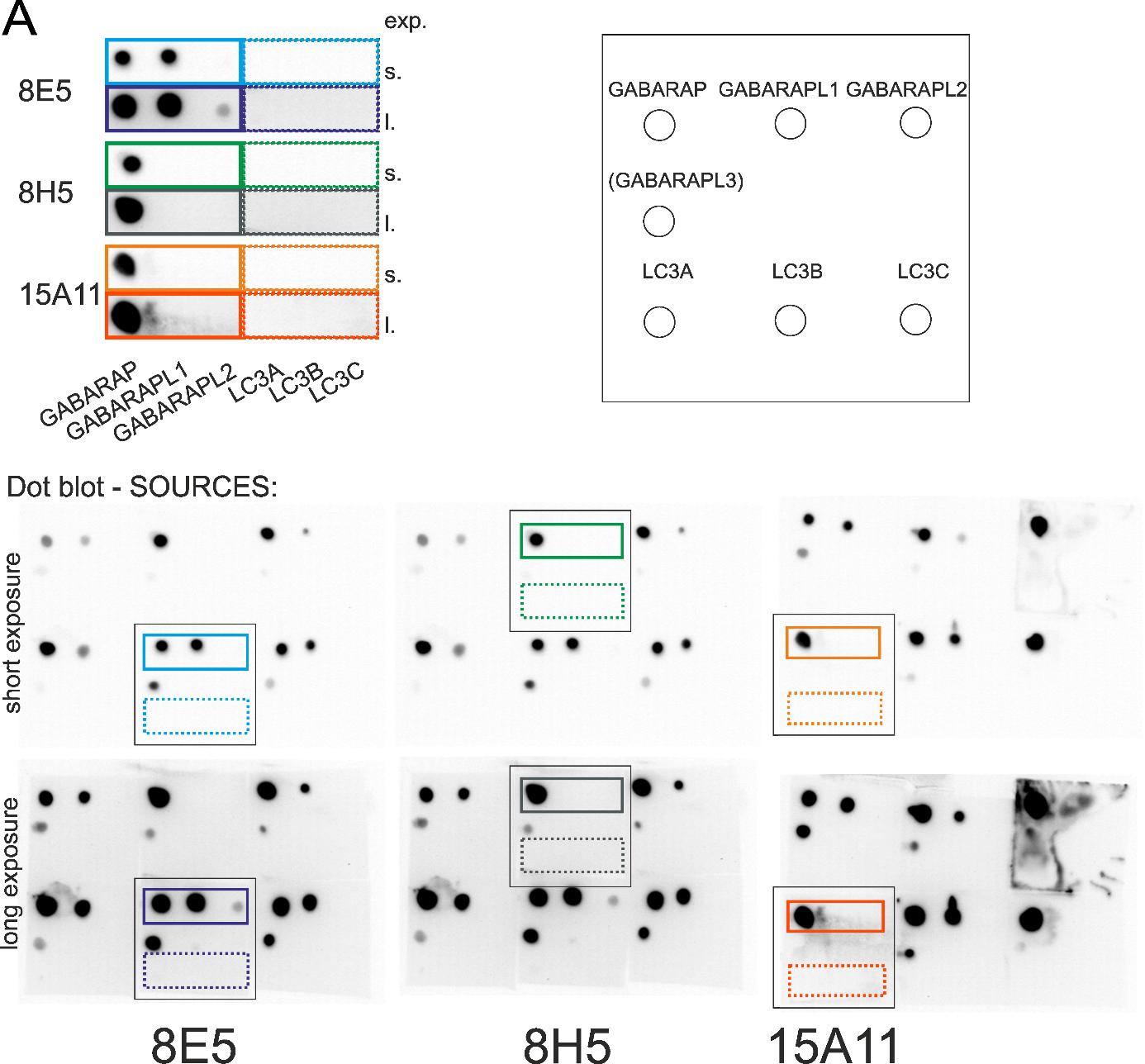
**

**
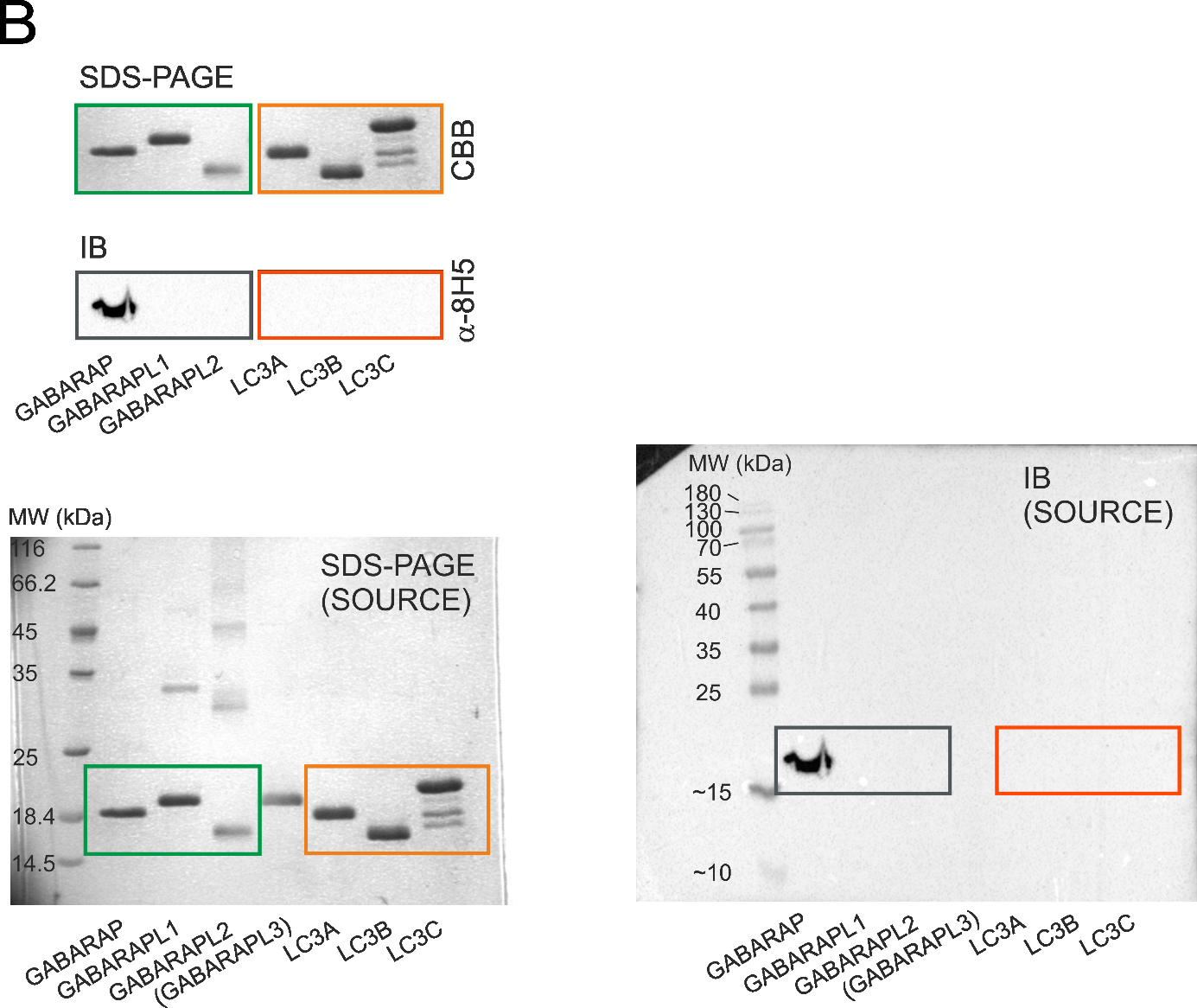
**

**
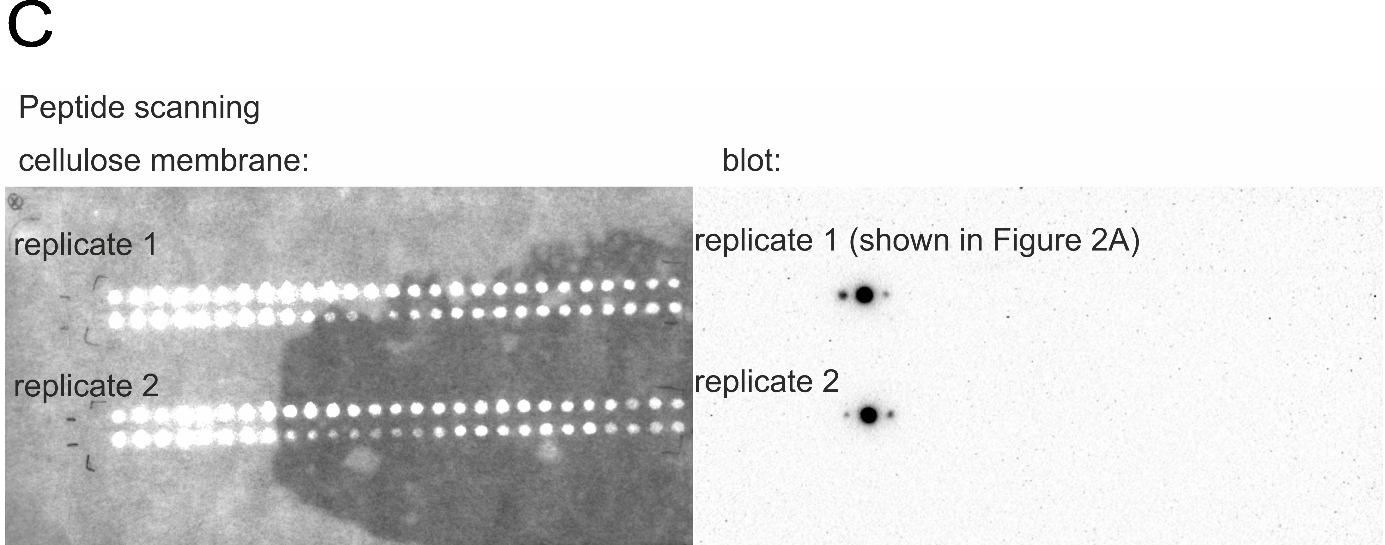
**

**
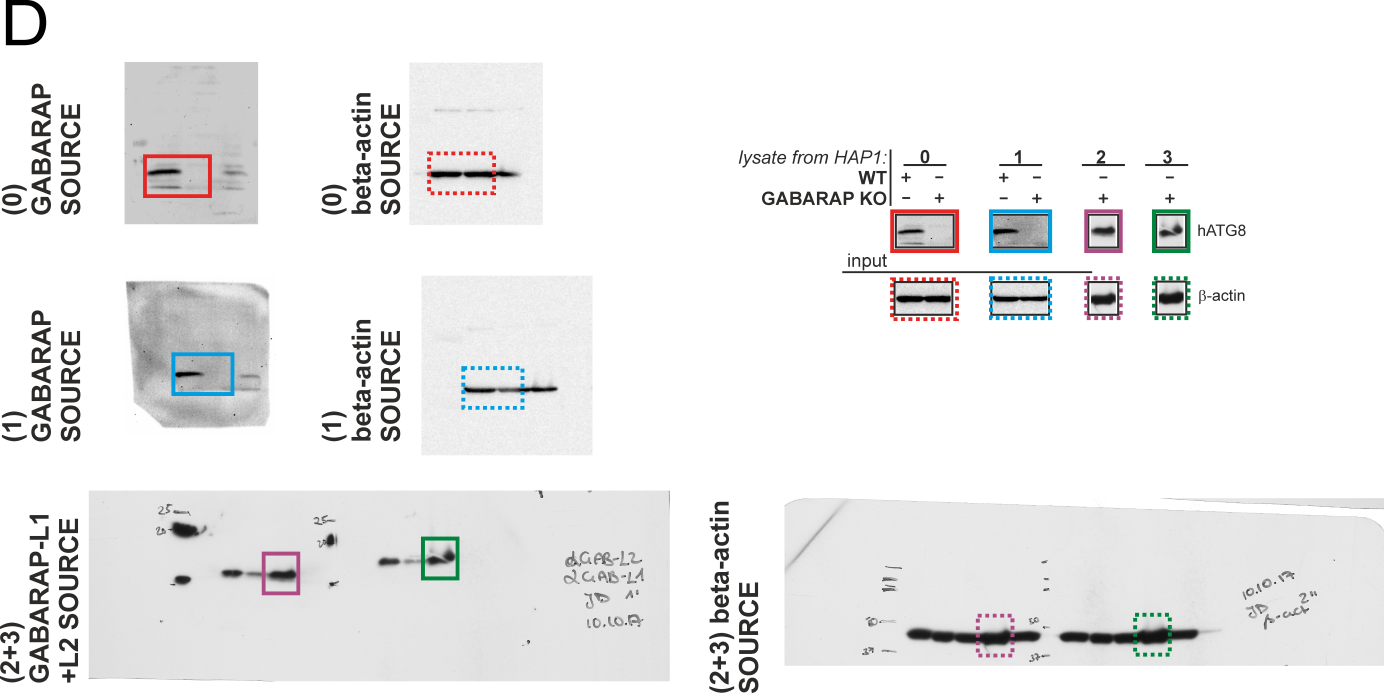
**


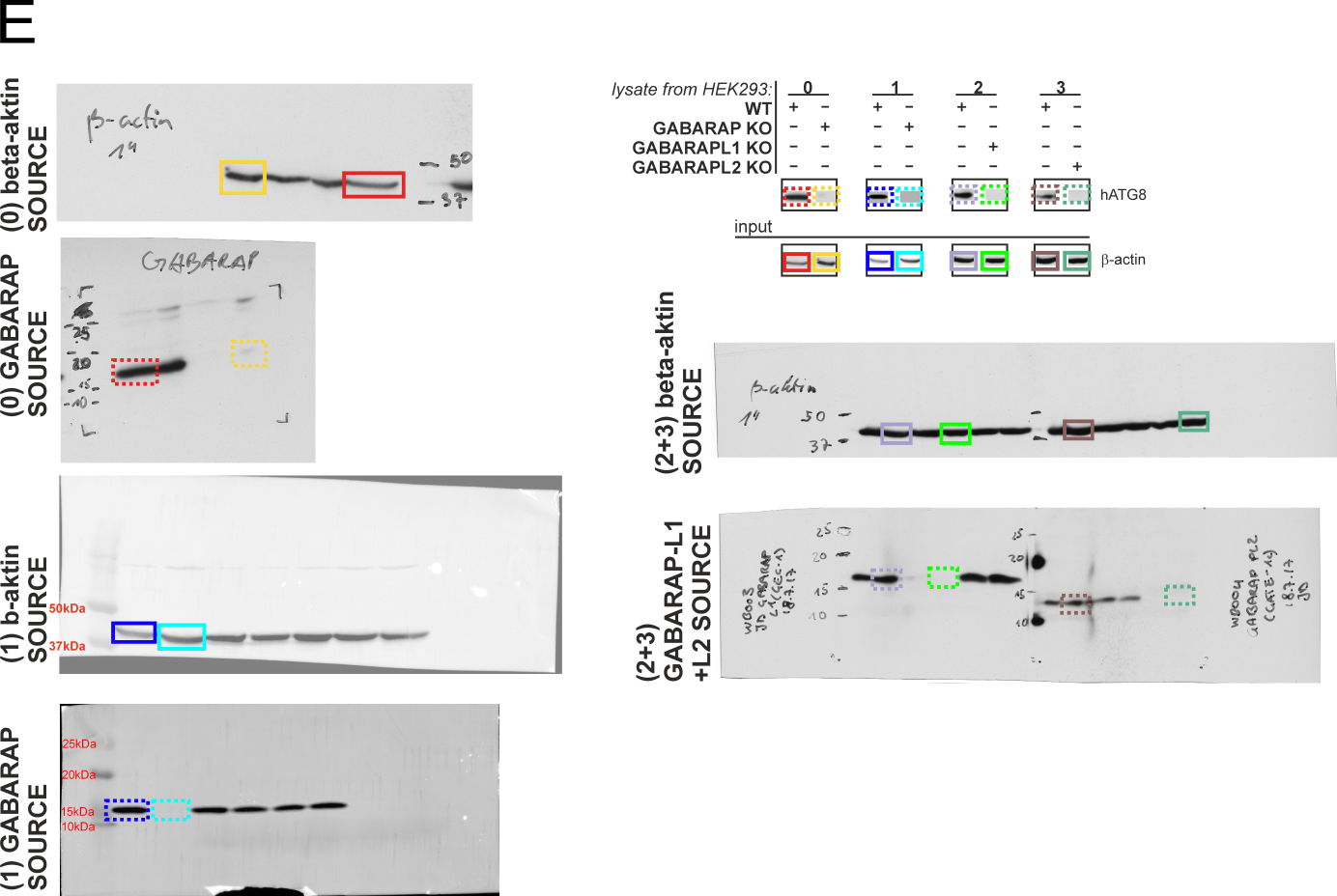


## Supplementary Figure S3. Full-length-blots. (A) Uncropped dot blots related to Figure 1A. Note: Each individual exposure includes six blots incubated with one of six different hybridoma supernatants each. Only those incubated with GABARAP-8E5, -8H5 and -15A11 containing supernatants are highlighted. Beside purified GABARAP, -L1, -L2, LC3A, -B and –C the hybridoma supernatants were also tested for their reactivity with GABARAPL3. Because GABARAPL3 is assumed to be a “pseudogene”, it was excluded during further analysis. (B) Uncropped SDS-PAGE and western blot membrane related to Figure 1C. (C) Uncropped cellulose membrane shown in Figure 2A used for epitope mapping of GABARAP-8H5. Transillumination and blot are given for both replicates. (D, E) Uncropped western blot membranes related to Supplementary Figure S1A, B.

## Supplementary Figure S4


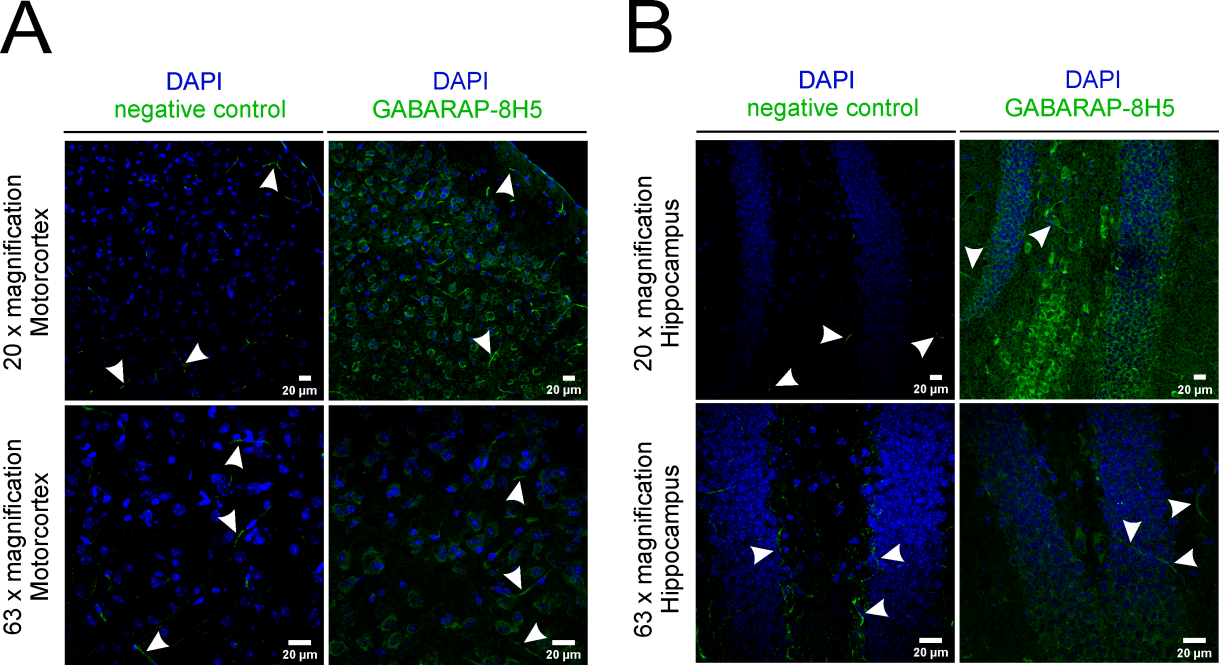


**Supplementary Figure S4. Performance of anti-GABARAP (8H5) antibody during immunofluorescent analysis of mouse brain slices.** Representative immunofluorescence images of motorcortex (A) or the hippocampal regions (B) of 20 weeks old mice stained with a goat anti-rat IgG+IgM (H&L) Alexa Fluor 488 secondary antibody in the absence (left panels) or presence of anti-GABARAP (8H5) primary antibody (right panels). All residual staining visible in the left panels is considered as non-specific, and likely represents blood vessels (white arrowheads). In the presence of anti-GABARAP (8H5) antibody a clear intra-cellular staining occurs (right panels). Interestingly, an antigen retrieval step as specified in the methods section was obligatory for positive staining results. Note that mouse and human GABARAP sequences display 100 % conservation. Counterstaining was performed with DAPI.

## Supplementary Figure S5


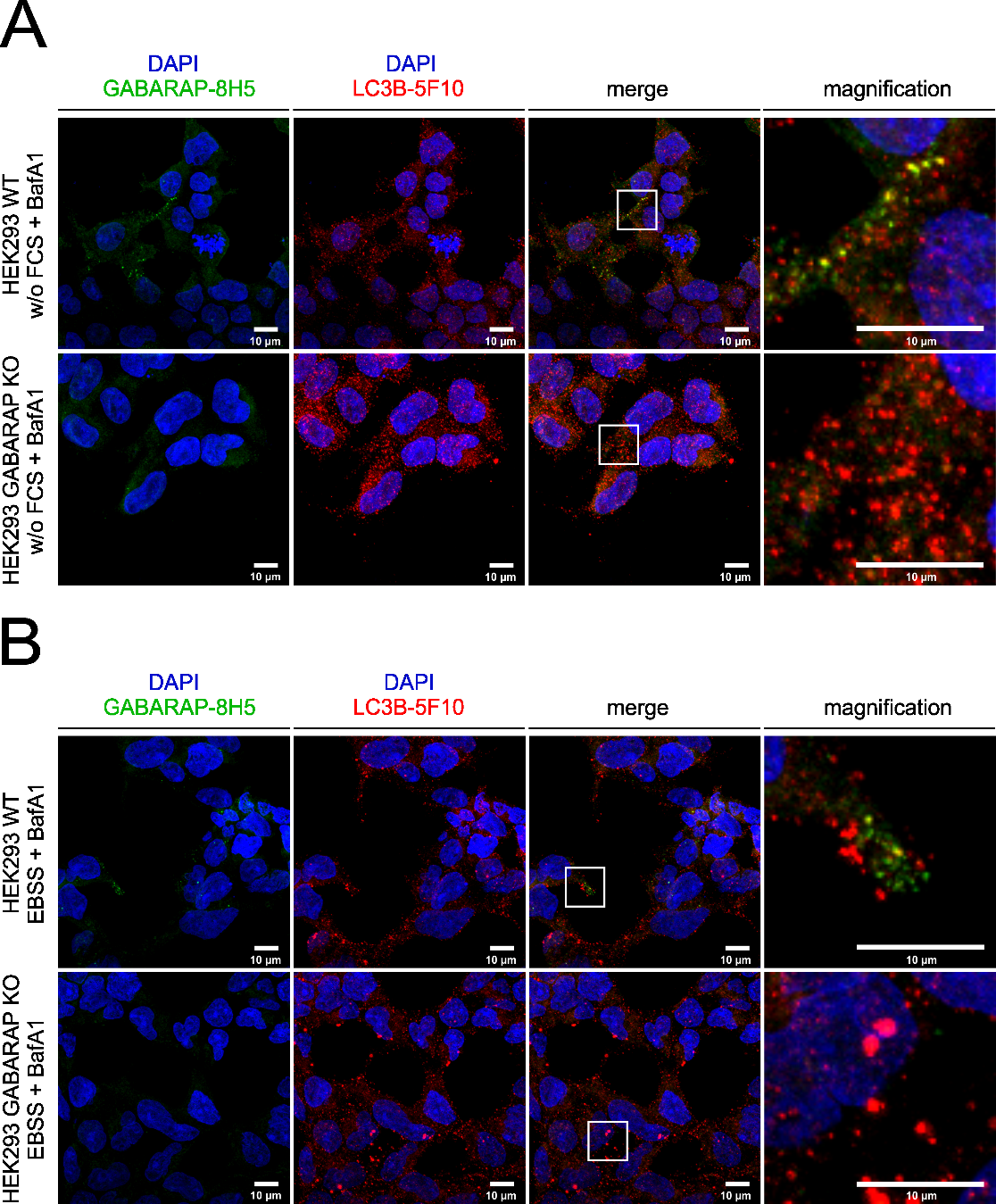


**Supplementary Figure S5. Performance of anti-GABARAP (8H5) in HEK293 cells.** HEK293 wildtype and GABARAP KO cells were incubated for 3 h in growth factor depleted medium (A) or in EBSS (B) both including 100 nM BafA1 each. Cells were fixed with 4 % PFA and immunolabeled with anti-GABARAP (8H5) and anti-LC3B (5F10) primary antibodies in combination with goat anti-rat IgG+IgM (H&L) Alexa Fluor 488 and anti-mouse IgG Alexa Fluor 647 secondary antibodies, respectively. Colocalization of LC3B (red) and GABARAP (green) is indicated by yellow puncta. Nuclei were counterstained with DAPI.

## Supplementary Figure S6


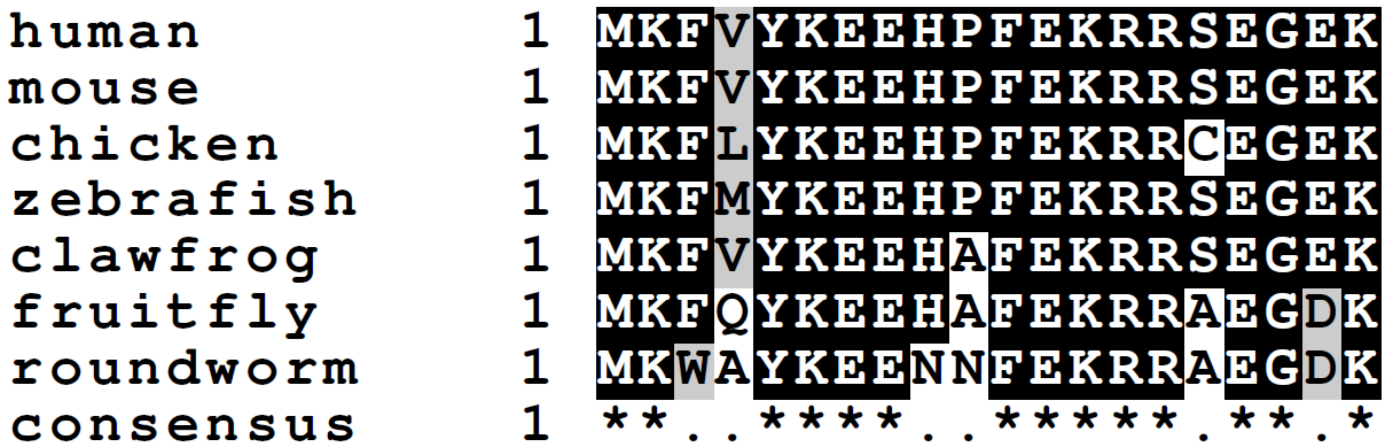


**Supplementary Figure S6. The epitope for anti-GABARAP (8H5) antibody shows high conservation between GABARAP proteins from organisms of diverse phylogenetic classes**. Aligned are the GABARAP residues 1 to 20 of human GABARAP with those of selected vertebrate, arthropode and nemathode orthologs using Boxshade 3.2.3. UniProtKB accession numbers of the entries used are: O95166 (Homo sapiens); Q9DCD6 (Mus musculus); A0A1L1RN80 (Gallus gallus); Q6PSS4 (Danio rerio); Q6NUG7 (Xenopus laevis); Q9W2S2 (Drosophila melanogaster; protein name: Atg8a); Q09490 (Caenorhabditis elegans; protein name: lgg-1).

**Supplementary Table S1. Genotyping primers for sequencing analysis the HEK293 knockout cell lines.**

| **Gene** | **Exon** | **Primer direction** | **Primer sequence**  **(5’ to 3’)** | **PCR product**  **(bp)** |
| --- | --- | --- | --- | --- |
| *GABARAP* | 1 | forward | GGGTTGGTGAATAGGGAAGTGG | 392 |
| *GABARAP-L1* | 2 | forward | TGCAGCTATAACCTCATGAAGCC | 400 |
| *GABARAP-L2* | 2 | forward | CTTGCTGGGAGCTAGTAGGG | 402 |
| *GABARAP* | 1 | reverse | CACTCCTTTCATCCTGGGTCC | 392 |
| *GABARAP-L1* | 2 | reverse | ACTCCAGAGCATCCCACTCA | 400 |
| *GABARAP-L2* | 2 | reverse | TGAGGCACCCTGAACAGCA | 402 |

Supplementary Table S2. CRISPR sequence details and genotyping results of the knockout cell lines used.

| **Gene symbol** | **Uniprot** | **Gene ID/**  **location** | **Targeting strategy** | **CRISPR gRNA (PAM)** | **Main clone** | **unique alleles** | **Mutation** | **Protein impact** |
| --- | --- | --- | --- | --- | --- | --- | --- | --- |
| *GABARAP* | O95166 | 11337/NC_000017.11 | first exon | GGATCTTCTCGCCCTCAGAGCGG | C2 | 1 | c.[152_153insT] | p.[fs*0] |
| *GABARAP-L1* | Q9H0R8 | 23710/NC_000012.12 | second exon | AGAGAAGGCTCCAAAAGCCAGGG | C10 | 2 | c.[352_357del];[353_356] | p.[K38Tfs*10];[K38Nfs*3] |
| *GABARAP-L2* | P60520 | 11345/NC_000016.1 | second exon | TCCCACAGAACACAGATGCGTGG | #8 | 1 | c.[179_180insT] | p.[C15Lfs*27] |

Formatting of indels detected in the knockout cell lines (Mutation column) and their resulting proteins (Protein impact column) is according to Human Genome Variation Society (http://varnomen.hgvs.org/). Mutation positions are determined in respect to the canonical isoform annotated in Uniprot, if more than one form exists. The numbers after the asterisks represent the number of amino acids present from the first amino acid changed to the next sequential stop codon. del, deletion; ins, insertion; c., coding DNA; p., protein; fs, frame shift; *, stop codon.

Supplementary Table S3. Antibodies used throughout this study.

| **Antibody** | **Company** | **Species** | **Catalog number**  **(antigen used for antibody production)** |
| --- | --- | --- | --- |
| primary Abs: |  |  |  |
| anti-GABARAP clone 8H5 | in-house | rat | (GST-hGABARAP_aa1-117) |
| anti-LC3B clone 5F10 | Nanotools | mouse | 0231-100  (synthetic peptide from N-terminus of hLC3B) |
| anti-GABARAP pAb | Proteintech | rabbit | 18723-1-AP  (GABARAP full-length protein) |
| anti-GABARAP clone E1J4E | Cell Signaling | rabbit | 13733  (synthetic peptide surrounding R40 of hGABARAP) |
| anti-GABARAP pAb clone RB11846 | Abgent/antibodies-online | rabbit | AP1821a/ABIN388564  (synthetic peptide between 1-31 aa from the N-terminal region of GABARAP) |
| anti-GABARAPL1 clone D5R9Y | Cell Signaling | rabbit | 26632  (synthetic peptide corresponding to residues near the N-terminus of hGABARAPL1) |
| anti-GABARAPL2 clone D1W9T | Cell Signaling | rabbit | 14256  (synthetic peptide corresponding to residues near the C-terminus of hGABARAPL2) |
| secondary Abs: |  |  |  |
| anti-rat IgG2a-HRP | in-house | mouse | (rat IgG 2a) |
| anti-rat-IgG-HRP | Sigma | goat | A9037 |
| anti-rat-IgG Alexa Fluor 488 | Jackson ImmunoResearch | goat | 112-545-068 |
| anti-rat-IgG Cy5 | Jackson ImmunoResearch | donkey | 712-175-153 |
| anti-mouse IgG Alexa Fluor 647 | Abcam | goat | ab150115 |
| anti-rabbit IgG Alexa  Fluor 555  anti-rabbit IgG Alexa Fluor 647 | Abcam  Abcam | donkey  donkey | ab150074  ab150075 |

## Additional References

1. Ran, F.A., et al. Genome engineering using the CRISPR-Cas9 system. *Nat Protoc* **8**, 2281-308, doi:10.1038/nprot.2013.143 (2013).
2. Dehairs, J., et al. CRISP-ID: decoding CRISPR mediated indels by Sanger sequencing. *Sci Rep* **6**, 28973, doi: 10.1038/srep28973 (2016).
3. Etard, C., et al. Tracking of Indels by DEcomposition is a Simple and Effective Method to Assess Efficiency of Guide RNAs in Zebrafish. *Zebrafish* **14**, 586-588, doi: 10.1089/zeb.2017.1454 (2017).
